# Characteristics and risk factors for post-COVID breathlessness after hospitalisation for COVID-19

**DOI:** 10.1183/23120541.00274-2022

**Published:** 2022-02-09

**Authors:** Luke Daines, Bang Zheng, Omer Elneima, Ewen Harrison, Nazir I. Lone, John R. Hurst, Jeremy S. Brown, Elizabeth Sapey, James D. Chalmers, Jennifer K. Quint, Paul Pfeffer, Salman Siddiqui, Samantha Walker, Krisnah Poinasamy, Hamish McAuley, Marco Sereno, Aarti Shikotra, Amisha Singapuri, Annemarie B. Docherty, Michael Marks, Mark Toshner, Luke S. Howard, Alex Horsley, Gisli Jenkins, Joanna C. Porter, Ling-Pei Ho, Betty Raman, Louise V. Wain, Christopher E. Brightling, Rachael A. Evans, Liam G. Heaney, Anthony De Soyza, Aziz Sheikh

**Affiliations:** 1 Usher Institute, University of Edinburgh, UK; 2 The Institute for Lung Health, Leicester NIHR Biomedical Research Centre, University of Leicester, UK; 3 UCL Respiratory, University College London, UK; 4 Centre for Translational Inflammation Research, University of Birmingham, UK; 5 School of Medicine, University of Dundee, UK; 6 National Heart & Lung Institute, Imperial College London, UK; 7 Barts Health NHS Trust and Queen Mary University of London, UK; 8 Asthma UK and British Lung Foundation, UK; 9 Clinical Research Department, London School of Hygiene & Tropical Medicine, UK; 10 Heart Lung Research Institute, Department of Medicine, University of Cambridge, UK; 11 Hammersmith Hospital, Imperial College Healthcare NHS Trust, UK; 12 Division of Infection, Immunity and Respiratory Medicine, University of Manchester, UK; 13 MRC Weatherall Institute of Molecular Medicine, Oxford University; 14 Radcliffe Department of Medicine, University of Oxford, UK; 15 Department of Health Sciences and NIHR Leicester Biomedical Research Centre, University of Leicester, UK; 16 Centre for Experimental Medicine, Queen's University Belfast, UK; 17 Population Health Science Institute, Newcastle University, UK

## Abstract

**Background:**

Persistence of respiratory symptoms—particularly breathlessness—after acute COVID-19 infection has emerged as a significant clinical problem. We aimed to characterise and identify risk factors for patients with persistent breathlessness following COVID-19 hospitalisation.

**Methods:**

PHOSP-COVID is a multi-centre prospective cohort study of UK adults hospitalised for COVID-19. Clinical data were collected during hospitalisation and at a follow-up visit. Breathlessness was measured by a numeric rating scale of 0–10. We defined post-COVID breathlessness as an increase in score of 1 or more compared to the pre-COVID-19 level. Multivariable logistic regression was used to identify risk factors, and to develop a prediction model for post-COVID breathlessness.

**Results:**

We included 1226 participants (37% female, median age 59 years, 22% mechanically ventilated). At a median five months after discharge, 50% reported post-COVID breathlessness. Risk factors for post-COVID breathlessness were socio-economic deprivation (adjusted odds ratio, 1.67; 95% confidence interval, 1.14–2.44), pre-existing depression/anxiety (1.58; 1.06–2.35), female sex (1.56; 1.21–2.00) and admission duration (1.01; 1.00–1.02). Black ethnicity (0.56; 0.35–0.89) and older age groups (0.31; 0.14–0.66) were less likely to report post-COVID breathlessness. Post-COVID breathlessness was associated with worse performance on the shuttle walk test and forced vital capacity, but not with obstructive airflow limitation. The prediction model had fair discrimination (concordance-statistic 0.66; 0.63–0.69), and good calibration (calibration slope 1.00; 0.80–1.21).

**Conclusions:**

Post-COVID breathlessness was commonly reported in this national cohort of patients hospitalised for COVID-19 and is likely to be a multifactorial problem with physical and emotional components.

This is a PDF-only article. Please click on the PDF link above to read it.

