# Characteristics and risk factors for post-COVID-19 breathlessness after hospitalisation for COVID-19

**DOI:** 10.1183/23120541.00274-2022

**Published:** 2023-02-20

**Authors:** Luke Daines, Bang Zheng, Omer Elneima, Ewen Harrison, Nazir I. Lone, John R. Hurst, Jeremy S. Brown, Elizabeth Sapey, James D. Chalmers, Jennifer K. Quint, Paul Pfeffer, Salman Siddiqui, Samantha Walker, Krisnah Poinasamy, Hamish McAuley, Marco Sereno, Aarti Shikotra, Amisha Singapuri, Annemarie B. Docherty, Michael Marks, Mark Toshner, Luke S. Howard, Alex Horsley, Gisli Jenkins, Joanna C. Porter, Ling-Pei Ho, Betty Raman, Louise V. Wain, Christopher E. Brightling, Rachael A. Evans, Liam G. Heaney, Anthony De Soyza, Aziz Sheikh

**Affiliations:** 1 Usher Institute, University of Edinburgh, Edinburgh, UK; 2 The Institute for Lung Health, Leicester NIHR Biomedical Research Centre, University of Leicester, Leicester, UK; 3 UCL Respiratory, University College London, London, UK; 4 Centre for Translational Inflammation Research, University of Birmingham, Birmingham, UK; 5 School of Medicine, University of Dundee, Dundee, UK; 6 National Heart and Lung Institute, Imperial College London, London, UK; 7 Barts Health NHS Trust and Queen Mary University of London, London, UK; 8 Asthma + Lung UK, London, UK; 9 Clinical Research Department, London School of Hygiene and Tropical Medicine, London, UK; 10 Heart Lung Research Institute, Department of Medicine, University of Cambridge, Cambridge, UK; 11 Hammersmith Hospital, Imperial College Healthcare NHS Trust, London, UK; 12 Division of Infection, Immunity and Respiratory Medicine, University of Manchester, Manchester, UK; 13 MRC Weatherall Institute of Molecular Medicine, Oxford University, Oxford, UK; 14 Radcliffe Department of Medicine, University of Oxford, Oxford, UK; 15 Department of Health Sciences and NIHR Leicester Biomedical Research Centre, University of Leicester, Leicester, UK; 16 Centre for Experimental Medicine, Queen's University Belfast, Belfast, UK; 17 Population Health Science Institute, Newcastle University, Newcastle, UK; 18 Members of the PHOSP-COVID Study Collaborative Group are listed in the supplementary material

## Abstract

**Background:**

Persistence of respiratory symptoms, particularly breathlessness, after acute coronavirus disease 2019 (COVID-19) infection has emerged as a significant clinical problem. We aimed to characterise and identify risk factors for patients with persistent breathlessness following COVID-19 hospitalisation.

**Methods:**

PHOSP-COVID is a multicentre prospective cohort study of UK adults hospitalised for COVID-19. Clinical data were collected during hospitalisation and at a follow-up visit. Breathlessness was measured by a numeric rating scale of 0–10. We defined post-COVID-19 breathlessness as an increase in score of ≥1 compared to the pre-COVID-19 level. Multivariable logistic regression was used to identify risk factors and to develop a prediction model for post-COVID-19 breathlessness.

**Results:**

We included 1226 participants (37% female, median age 59 years, 22% mechanically ventilated). At a median 5 months after discharge, 50% reported post-COVID-19 breathlessness. Risk factors for post-COVID-19 breathlessness were socioeconomic deprivation (adjusted OR 1.67, 95% CI 1.14–2.44), pre-existing depression/anxiety (adjusted OR 1.58, 95% CI 1.06–2.35), female sex (adjusted OR 1.56, 95% CI 1.21–2.00) and admission duration (adjusted OR 1.01, 95% CI 1.00–1.02). Black ethnicity (adjusted OR 0.56, 95% CI 0.35–0.89) and older age groups (adjusted OR 0.31, 95% CI 0.14–0.66) were less likely to report post-COVID-19 breathlessness. Post-COVID-19 breathlessness was associated with worse performance on the shuttle walk test and forced vital capacity, but not with obstructive airflow limitation. The prediction model had fair discrimination (concordance statistic 0.66, 95% CI 0.63–0.69) and good calibration (calibration slope 1.00, 95% CI 0.80–1.21).

**Conclusions:**

Post-COVID-19 breathlessness was commonly reported in this national cohort of patients hospitalised for COVID-19 and is likely to be a multifactorial problem with physical and emotional components.

## Abstract 2

**Socioeconomic deprivation, pre-existing depression/anxiety, female sex and longer admission duration were risk factors for persistent breathlessness in patients assessed 5 months after hospitalisation for COVID-19** https://bit.ly/3TnzMq1

## Introduction

Coronavirus disease 2019 (COVID-19) continues to have a huge impact internationally [1]. The post-acute COVID-19 syndrome (also known as “long COVID”) usually occurs 3 months from the onset of COVID-19, with symptoms that last for ≥2 months and cannot be explained by an alternative diagnosis [2]. The term “long COVID” may also be used to refer to ongoing symptomatic COVID-19 occurring between 4 and 12 weeks after acute COVID-19 infection [3]. With increasing understanding of the debilitating longer-term effects of COVID-19 [4–7], characterising and being able to predict which individuals will suffer from long COVID is a policy priority [8].

Breathlessness is one of the most common and burdensome symptoms reported by individuals, forming part of a complex of respiratory symptoms observed in long COVID [9]. The prevalence of persistent breathlessness in hospitalised and non-hospitalised patients after acute COVID-19 is estimated to be between 26% and 39% [10–14]. Breathlessness is understood as a multidimensional disease concept with different underlying physiological mechanisms including respiratory and cardiovascular diseases, deconditioning, being overweight and emotional factors such as anxiety [15, 16].

In a community-based sample investigating the persistence of symptoms 12 weeks after acute COVID-19, a respiratory-predominant symptom cluster including breathlessness, chest tightness and chest pain was identified [17]. Within this respiratory cluster, a higher proportion of individuals were obese, cigarette smokers, had more comorbidities and considered their acute COVID-19 symptoms severe [17]. In a single-site study of 478 hospital survivors, new-onset dyspnoea was more likely in younger patients, those treated in the intensive therapy unit (ITU) and those with pulmonary embolism [18]; yet another smaller study found no association with dyspnoea at 3 months and ITU admission [19]. A further single-site study of 119 adults hospitalised with severe COVID-19 pneumonia found that failure to return to pre-COVID-19 breathlessness a median 61 days after discharge was associated with comorbid obstructive lung disease, and high scores on anxiety, depression or post-COVID-19 functional status screening, but not ITU admission or inpatient pulmonary embolism [20].

In this study, we sought to estimate the frequency of and characterise risk factors for persisting breathlessness using a multicentre cohort of patients who were discharged following hospitalisation for COVID-19. A secondary aim was to derive a prediction model to identify individuals most at risk of new or worsening breathlessness post-hospitalisation for COVID-19.

## Methods

### Study design, setting and population

PHOSP-COVID is a multicentre prospective cohort study of adults discharged from one of 53 National Health Service (NHS) hospitals in the UK following admission for COVID-19. Data were collected during hospital admission and at a research visit, between 1 and 8 months after discharge (depending on participant and investigator availability), from clinical health records, and supplemented by questionnaires, clinical and research samples and additional clinical assessments. Participants aged ≥18 years who were discharged from hospital following inpatient treatment for COVID-19 based on a positive reverse transcriptase (RT)-PCR test for severe acute respiratory syndrome coronavirus 2 (SARS-CoV-2) or clinician diagnosis (if there was a high index of suspicion and testing was either unavailable or considered inaccurate) were included. Individuals were excluded if they attended the emergency department but were not admitted to hospital or had an existing condition with a life expectancy <6 months. Recruitment occurred between August 2020 and November 2021. Here, we report on the patients who provided data for breathlessness both before COVID-19 and at their first research assessment, before January 2022.

### Data collection and outcome

Patient characteristics prior to admission, during hospitalisation and at the research visit were considered. We included patient demographics, patient-reported past medical history, number of comorbidities, body mass index (BMI) and smoking status. Hospital admission data included the level of respiratory support received (categorised based on the World Health Organization clinical progression scale) (supplementary table S1) [21], length of stay, treatments and complications during hospitalisation. At the research visit, patient-reported outcomes were collected using the General Anxiety Disorder-7 Questionnaire (GAD-7) [22], Patient Health Questionnaire-9 (PHQ-9) [23] and Post Traumatic Stress Disorder Checklist (PCL-5) [24]. Results from clinical tests included full blood count, C-reactive protein, N-terminal pro-B-type natriuretic peptide or B-type natriuretic peptide, lung function tests, and the incremental shuttle walk test (ISWT).

Lung function testing was limited at certain recruiting sites due to COVID-19 restrictions [25]. Forced expiratory volume in 1 s (FEV_1_) and forced vital capacity (FVC) were measured in accordance with American Thoracic Society/European Respiratory Society criteria [26] and used to calculate the FEV_1_/FVC ratio. Airflow obstruction was defined by an FEV_1_/FVC ratio less than the lower limit of normal (LLN). Transfer capacity of the lung for carbon monoxide (*T*_LCO_) and carbon monoxide transfer coefficient (*K*_CO_) were obtained using the best of two readings. Percent predicted and LLN were calculated using Global Lung Function Initiative equations [27, 28].

At the research visit, participants reported their perceived breathlessness at the time of the visit and recalled their level of breathlessness before developing COVID-19 using a Patient Symptom Questionnaire (PSQ), a numeric rating scale between 0 and 10 (supplementary figure S1). The availability of the PSQ breathlessness score at the time of the research visit and before COVID-19 allowed a new variable to be created, which we defined as “post-COVID-19 breathlessness”; this was used as our primary outcome. In line with Johnson *et al.* [29], we took the minimum clinically important difference for a change in breathlessness as 1 point on the 0–10 numeric rating scale. Thus, individuals who rated their breathlessness at the time of the research visit as at least 1 point greater than before developing COVID-19 (*i.e.* they reported new or worsening breathlessness compared to baseline), were categorised as having post-COVID-19 breathlessness. Sensitivity analyses were performed using breathlessness reported at the time of the research visit based on 1) PSQ and 2) Dyspnoea-12 (which was only reported at the research visit) [30].

### Statistical analysis

We used descriptive statistics to describe participant characteristics. Continuous variables were presented as means and standard deviations or medians and interquartile ranges, as appropriate. Binary and categorical variables were presented as counts and percentages.

For the primary outcome, we report univariable and multivariable logistic regression with and without imputed data. Continuous explanatory variables were checked for linearity compared with the dependent variable and included with a quadratic term when necessary. Explanatory variables were assessed for multicollinearity. Explanatory variables collected at the research visit were not included in the multivariable model for two reasons. First, the model was intended to make predictions for breathlessness using data available at hospital discharge. Second, due to the multisite nature of the study, certain variables (such as lung function) were likely to be missing at specific sites in a systematic manner, making imputation of these variables inappropriate. Explanatory variables were added to the model manually following initial descriptive analysis (though not based on a p-value threshold) and in consultation with the expert clinical group. Final model selection was based on a criterion-based approach intending to minimise the Akaike Information Criteria (AIC) and maximise the concordance statistic (C-statistic). First-order interactions were checked and included if influential. Under the assumption that missing values within variables were missing at random, we used multiple imputation by chained equations to create 20 datasets each with 10 iterations based on the following variables: sex at birth, age at admission (as a factor), ethnicity, socioeconomic status determined using the Index of Multiple Deprivation (IMD) expressed as quintiles, BMI, number of comorbidities, pre-existing respiratory disease, pre-existing depression or anxiety, admission duration, level of respiratory support, and post-COVID-19 breathlessness. Apparent performance measures of the prediction model were evaluated using the C-statistic, expected/observed number of events (E/O), calibration slope (each calculated using the median from the 20 imputed datasets) and calibration plot (evaluated in the first imputed dataset). To investigate differences between individuals according to the severity of post-COVID-19 breathlessness, multinomial modelling was used in the imputed dataset (supplementary table S2). To assess the associations between clinical measures during the research visit and post-COVID-19 breathlessness, separate multivariable logistic regression models were fitted, adjusting for age, sex, ethnicity and IMD.

We used R (version 3.6.3) for all statistical analysis.

## Results

### Participants

**FIGURE 1:**
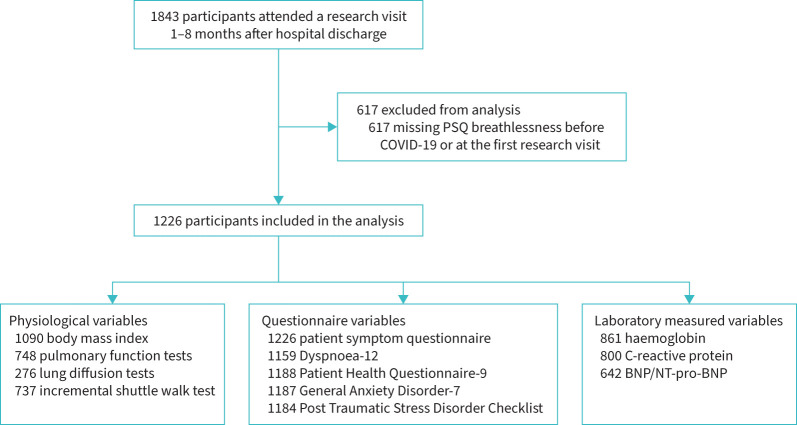
Flow diagram of participants. PSQ: patient symptom questionnaire; BNP: B-type natriuretic peptide; NT-pro-BNP: N-terminal pro-BNP.

**FIGURE 2:**
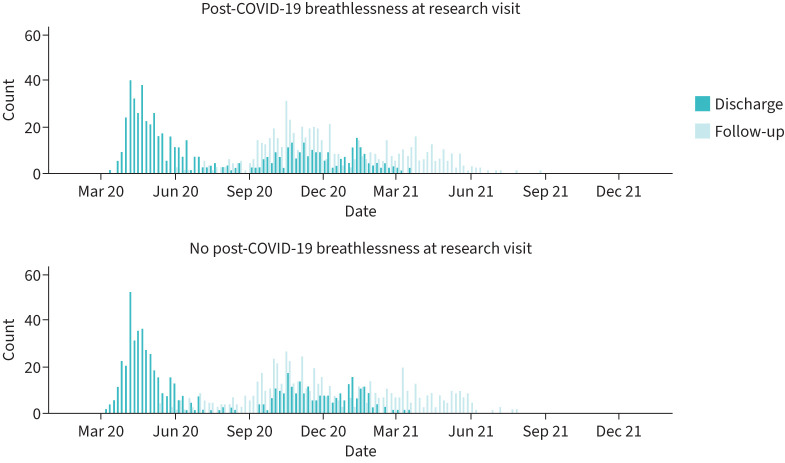
Dates of discharge and research visit for the 1226 study participants.

1843 participants attended a research visit between 1 and 8 months after discharge, of whom 617 had no data for breathlessness and were excluded (figure 1). There were no clear differences between included and excluded participants (supplementary table S3). Of the 1226 participants included in this analysis, 458 (37%) were female and the median age was 59 years (range 21–89 years). 873 (71%) were of white ethnicity (table 1). Median admission duration was 8 days (interquartile range 4–17 days) (table 2). Of those with data for RT-PCR, 1039 (85%) had a positive result. 270 (22%) patients required the highest level of respiratory support (*i.e.* invasive mechanical ventilation). 714 (58%) participants were discharged between March and July 2020 (figure 2). There was a higher proportion of missingness for the clinical tests at the research visit (table 3) compared with data collected during hospitalisation.

**TABLE 1:** Patient characteristics

	**Total, N (%)**	**Post-COVID-19 breathlessness**		**Total**
		**No**	**Yes**	
**Total, N (%)**		611 (49.8)	615 (50.2)	1226
**Age at admission, years**	1213 (98.9)			
<30		10 (1.6)	15 (2.4)	25 (2.0)
30–39		38 (6.2)	41 (6.7)	79 (6.4)
40–49		87 (14.2)	103 (16.7)	190 (15.5)
50–59		151 (24.7)	203 (33.0)	354 (28.9)
60–69		183 (30.0)	174 (28.3)	357 (29.1)
70–79		107 (17.5)	61 (9.9)	168 (13.7)
≥80		29 (4.7)	11 (1.8)	40 (3.3)
Data missing		6 (1.0)	7 (1.1)	13 (1.1)
**Sex at birth**	1226 (100.0)			
Male		416 (68.1)	352 (57.2)	768 (62.6)
Female		195 (31.9)	263 (42.8)	458 (37.4)
**Ethnicity**	1203 (98.1)			
White		422 (69.1)	451 (73.3)	873 (71.2)
South Asian		82 (13.4)	71 (11.5)	153 (12.5)
Black		52 (8.5)	40 (6.5)	92 (7.5)
Mixed		17 (2.8)	16 (2.6)	33 (2.7)
Other		27 (4.4)	25 (4.1)	52 (4.2)
Data missing		11 (1.8)	12 (2.0)	23 (1.9)
**Index of Multiple Deprivation**	1204 (98.2)			
1, most deprived		112 (18.3)	154 (25.0)	266 (21.7)
2		135 (22.1)	131 (21.3)	266 (21.7)
3		116 (19.0)	112 (18.2)	228 (18.6)
4		111 (18.2)	103 (16.7)	214 (17.5)
5, least deprived		127 (20.8)	103 (16.7)	230 (18.8)
Data missing		10 (1.6)	12 (2.0)	22 (1.8)
**BMI, kg·m^−2^, mean±sd**	1090 (88.9)	31.4±7.1	32.7±7.1	32.0±7.1
**Smoking**	1213 (98.9)			
Never-smokers		350 (57.3)	339 (55.1)	689 (56.2)
Ex-smokers		246 (40.3)	260 (42.3)	506 (41.3)
Current smokers		7 (1.1)	11 (1.8)	18 (1.5)
Data missing		8 (1.3)	5 (0.8)	13 (1.1)
**Number of comorbidities, median (IQR)**	1226 (100.0)	2.0 (0.0–3.0)	2.0 (1.0–4.0)	2.0 (0.0–3.0)
**Pre-existing cardiovascular condition**	1226 (100.0)			
No		329 (53.8)	350 (56.9)	679 (55.4)
Yes		282 (46.2)	265 (43.1)	547 (44.6)
**Pre-existing respiratory condition**	1226 (100.0)			
No		449 (73.5)	444 (72.2)	893 (72.8)
Yes		162 (26.5)	171 (27.8)	333 (27.2)
**Pre-existing depression or anxiety**	1207 (98.5)			
No		538 (88.1)	471 (76.6)	1009 (82.3)
Yes		66 (10.8)	132 (21.5)	198 (16.2)
Data missing		7 (1.1)	12 (2.0)	19 (1.5)
**Breathlessness before COVID-19, PSQ**	1226 (100.0)			
0		374 (61.2)	407 (66.2)	781 (63.7)
1–2		103 (16.9)	128 (20.8)	231 (18.8)
≥3		134 (21.9)	80 (13.0)	214 (17.5)

Data are presented as n (%) unless otherwise stated. BMI: body mass index; IQR: interquartile range; PSQ: Patient Symptom Questionnaire.

**TABLE 2:** Patient characteristics available during hospital admission

	**Total, N (%)**	**Post-COVID-19 breathlessness**		**Total**
		**No**	**Yes**	
**Total, N (%)**		611 (49.8)	615 (50.2)	1226
**Admission duration, days, median (IQR)**	1225 (99.9)	7.0 (4.0–14.0)	9.0 (4.0–22.0)	8.0 (4.0–17.0)
**SARS-CoV-2 PCR result**	1137 (92.7)			
Negative		47 (7.7)	51 (8.3)	98 (8.0)
Positive		522 (85.4)	517 (84.1)	1039 (84.7)
Data missing		42 (6.9)	47 (7.3)	89 (7.3)
**WHO clinical progression scale**	1226 (100.0)			
WHO class 3–4		110 (18.0)	113 (18.4)	223 (18.2)
WHO class 5		252 (41.2)	225 (36.6)	477 (38.9)
WHO class 6		136 (22.3)	120 (19.5)	256 (20.9)
WHO class 7–9		113 (18.5)	157 (25.5)	270 (22.0)
**Proning during mechanical ventilation**	1102 (89.9)			
No		466 (76.3)	426 (69.3)	892 (72.8)
Yes		87 (14.2)	123 (20.0)	210 (17.1)
Data missing		58 (9.5)	66 (10.7)	124 (10.1)
**Pulmonary embolism**	1146 (93.5)			
No		518 (84.8)	507 (82.4)	1025 (83.6)
Yes		56 (9.2)	65 (10.6)	121 (9.9)
Data missing		37 (6.1)	43 (7.0)	80 (6.5)
**Coronary thrombosis**	1140 (93.0)			
No		570 (93.3)	565 (91.9)	1135 (92.6)
Yes		<5	<5	5 (0.4)
Data missing				86 (7.0)
**Antibiotic therapy**	1187 (96.8)			
No		115 (18.8)	121 (19.7)	236 (19.2)
Yes		477 (78.1)	474 (77.1)	951 (77.6)
Data missing		19 (3.1)	20 (3.3)	39 (3.2)
**Systemic steroids, oral or *i.v.***	1144 (93.3)			
No		319 (52.2)	294 (47.8)	613 (50.0)
Yes		250 (40.9)	281 (45.7)	531 (43.3)
Data missing		42 (6.9)	40 (6.5)	82 (6.7)
**Therapeutic dose anticoagulation**	1150 (93.8)			
No		352 (57.6)	333 (54.1)	685 (55.9)
Yes		220 (36.0)	245 (39.8)	465 (37.9)
Data missing		39 (6.4)	37 (6.0)	76 (6.2)

Data are presented as n (%) unless otherwise stated. World Health Organization (WHO) clinical progression scale: not requiring continuous supplemental oxygen (levels 3–4); continuous supplemental oxygen only (level 5); continuous positive airway pressure ventilation, bilevel positive airway pressure or high-flow nasal oxygen (level 6); invasive mechanical ventilation, extra-corporeal membrane oxygenation and acute renal replacement therapy (levels 7–9). n<5 and related subtotals have been suppressed. IQR: interquartile range.

**TABLE 3:** Patient characteristics available at the research visit

	**Total, N (%)**	**Post-COVID-19 breathlessness**		**Total**	**OR (95% CI)**
		**No**	**Yes**		
**Total, N (%)**		611 (49.8)	615 (50.2)	1226	
**Discharge to review period, months**	1226 (100.0)	4.7 (3.4–6.0)	4.7 (3.3–6.0)	4.7 (3.4–6.0)	1.00 (1.00–1.00)
**Breathlessness at research visit, PSQ**	1226 (100.0)				
0		462 (75.6)	0 (0.0)	462 (37.7)	
1–2		70 (11.5)	143 (23.3)	213 (17.4)	
≥3		79 (12.9)	472 (76.7)	551 (44.9)	
**PHQ-9 total score**	1188 (96.9)	3.0 (1.0–8.0)	8.0 (3.0–13.0)	5.0 (2.0–11.0)	1.09 (1.07–1.12)
**GAD-7 total score**	1187 (96.8)	2.0 (0.0–6.0)	5.0 (1.0–11.0)	3.0 (0.0–8.0)	1.07 (1.05–1.09)
**PCL-5 total severity score**	1184 (96.6)	6.0 (2.0–15.0)	15.0 (5.0–31.2)	9.0 (3.0–23.0)	1.03 (1.02–1.04)
**CRP, mg·L^−1^**	800 (65.3)	4.0 (1.4–5.0)	4.0 (2.0–5.0)	4.0 (1.8–5.0)	1.01 (0.99–1.04)
**BNP/NT-pro-BNP above threshold**	642 (52.4)				0.65 (0.32–1.33)
No		293 (48.0)	304 (49.4)	597 (48.7)	
Yes		29 (4.7)	16 (2.6)	45 (3.7)	
**Haemoglobin, g·dL^−1^**	861 (70.2)	14.4 (13.3–15.2)	14.0 (13.1–15.0)	14.2 (13.2–15.2)	0.88 (0.79–0.98)
Males	537 (43.8)	14.7 (13.9–15.6)	14.6 (13.6–15.5)	14.7 (13.8–15.5)	0.90 (0.79–1.03)
Females	324 (26.4)	13.5 (12.8–14.3)	13.4 (12.6–14.0)	13.4 (12.7–14.1)	0.80 (0.65–0.99)
**ISWT distance, m**	737 (60.1)	440.0 (270.0–615.0)	350.0 (230.0–540.0)	380.0 (257.5–570.0)	0.91 (0.85–0.97)^#^
**ISWT, % predicted**	658 (53.7)	60.5 (42.0–81.9)	52.5 (35.1–71.2)	56.3 (37.9–75.9)	0.99 (0.98–1.00)
**Oxygen saturations post-ISWT, %**	727 (59.3)	96.0 (94.0–98.0)	96.0 (94.0–98.0)	96.0 (94.0–98.0)	0.98 (0.94–1.02)
**Borg leg fatigue score post-ISWT**	722 (58.9)	2.0 (0.5–3.0)	3.0 (2.0–4.0)	3.0 (1.0–4.0)	1.14 (1.06–1.23)
**FEV_1_, L**	748 (61.0)	2.8 (2.3–3.4)	2.7 (2.2–3.3)	2.8 (2.2–3.3)	0.96 (0.81–1.15)
**FEV_1_, % predicted**	683 (55.7)	93.9 (83.4–105.7)	89.9 (77.9–101.3)	91.7 (79.7–103.7)	1.00 (0.99–1.00)
**FEV_1_ <LLN**	683 (55.7)				1.61 (1.05–2.45)
No		274 (85.4)	284 (78.5)	558 (81.7)	
Yes		47 (14.6)	78 (21.5)	125 (18.3)	
**FVC, L**	746 (60.8)	3.6 (2.9–4.3)	3.3 (2.6–4.0)	3.5 (2.8–4.2)	0.70 (0.57–0.86)
**FVC, % predicted**	681 (55.5)	93.7 (83.0–105.4)	86.8 (74.5–98.5)	90.0 (78.2–102.4)	0.98 (0.97–0.99)
**FVC <LLN**	681 (55.5)				2.43 (1.60–3.70)
No		276 (86.2)	260 (72.0)	536 (78.7)	
Yes		44 (13.8)	101 (28.0)	145 (21.3)	
**FEV_1_/FVC ratio, %**	736 (60.0)	79.4 (73.9–84.0)	81.6 (77.3–86.0)	80.6 (76.0–85.5)	1.04 (1.02–1.06)
**FEV_1_/FVC <LLN**	673 (54.9)				0.58 (0.28–1.19)
No		295 (93.1)	342 (96.1)	637 (94.7)	
Yes		22 (6.9)	14 (3.9)	36 (5.3)	
***T*_LCO_, mmol·min^−1^·kPa^−1^**	272 (22.2)	7.6 (6.4–8.7)	6.8 (5.8–8.3)	7.3 (6.1–8.4)	0.94 (0.83–1.07)
***T*_LCO_, % predicted**	252 (20.6)	90.1 (78.6–102.7)	90.7 (74.2–104.2)	90.7 (76.8–103.2)	0.99 (0.99–1.00)
***T*_LCO_ <80% predicted**	252 (20.6)				1.48 (0.79–2.77)
No		86 (72.3)	90 (67.7)	176 (69.8)	
Yes		33 (27.7)	43 (32.3)	76 (30.2)	
***K*_CO_, mmol·min^−1^·kPa^−1^·L^−1^**	276 (22.5)	1.5 (1.3–1.6)	1.5 (1.2–1.7)	1.5 (1.3–1.6)	0.49 (0.18–1.29)
***K*_CO_, % predicted**	259 (21.1)	103.5 (92.6–108.7)	99.6 (87.4–112.3)	101.8 (89.2–110.1)	0.99 (0.97–1.00)
***K*_CO_ <80% predicted**	259 (21.1)				1.43 (0.53–3.88)
No		112 (92.6)	127 (92.0)	239 (92.3)	
Yes		9 (7.4)	11 (8.0)	20 (7.7)	

Data are presented as median (interquartile range) or n (%), unless otherwise stated. PSQ: Patient Symptom Questionnaire; PHQ-9: Patient Health Questionnaire-9; GAD-7: General Anxiety Disorder-7; PCL-5: Post Traumatic Stress Disorder Checklist; CRP: C-reactive protein; BNP: B-type natriuretic peptide; NT-pro-BNP: N-terminal pro-BNP; ISWT: incremental shuttle walk test; FEV_1_: forced expiratory volume in 1 s; LLN: lower limit of normal; FVC: forced vital capacity; *T*_LCO_: transfer capacity of the lung for carbon monoxide; *K*_CO_: carbon monoxide transfer coefficient. ^#^: refers to the risk of worsening breathlessness for each 100 m achieved.

### Main results

615 (50%) participants reported post-COVID-19 breathlessness at the research visit compared to their pre-COVID-19 baseline level, of whom 407 reported no breathlessness (PSQ 0) at baseline (table 1). Females were more likely to report post-COVID-19 breathlessness than males (57% *versus* 46%) (table 1 and supplementary figure S2). There was little difference between individuals with and without post-COVID-19 breathlessness in ethnicity (supplementary figure S3), smoking status, or number of comorbidities including the pre-existence of respiratory or cardiovascular diseases. However, the prevalence of pre-existing depression or anxiety was higher in the group with post-COVID-19 breathlessness (22% *versus* 11%) and those with post-COVID-19 breathlessness had a slightly higher BMI (mean 32.7 *versus* 31.4 kg·m^−2^). Individuals with post-COVID-19 breathlessness had longer hospital admission (median 9 *versus* 7 days), with little or no difference in the level of respiratory support required, medications (including corticosteroids) received or in-hospital complications (table 2).

**FIGURE 3:**
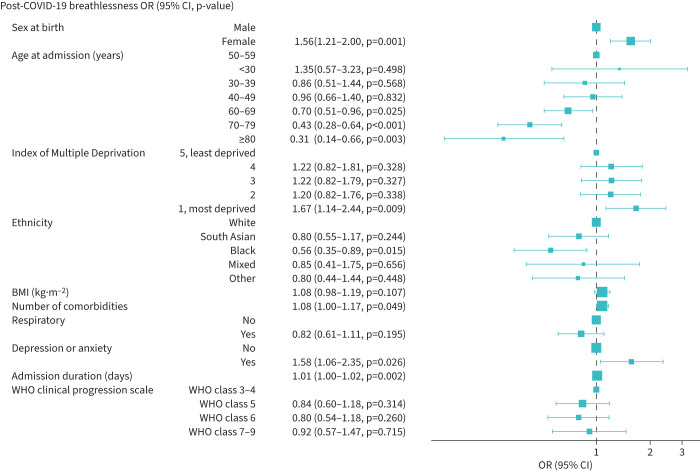
Multivariable logistic regression for post-COVID-19 breathlessness. BMI: body mass index; WHO: World Health Organization.

The multivariable logistic regression identified that post-COVID-19 breathlessness was associated with the most deprived quintile (adjusted OR 1.67, 95% CI 1.14–2.44) (table 4 and figure 3), pre-existing depression/anxiety (adjusted OR 1.58, 95% CI 1.06–2.35), female sex (adjusted OR 1.56, 95% CI 1.21–2.00) and admission duration (adjusted OR 1.01, 95% CI 1.00–1.02 per day). Individuals of Black ethnicity (adjusted OR 0.56, 95% CI 0.35–0.89) were less likely to report post-COVID-19 breathlessness. Compared to 50–59-year-olds, participants aged 60–69 years (adjusted OR 0.70, 95% CI 0.51–0.96), 70–79 years (adjusted OR 0.43, 95% CI 0.28–0.64) and ≥80 years (adjusted OR 0.31, 95% CI 0.14–0.66) were less likely to report post-COVID-19 breathlessness. The level of respiratory support received, pre-existing respiratory disease, number of comorbidities and BMI were not associated with post-COVID-19 breathlessness.

**TABLE 4:** Multivariable logistic regression for post-COVID-19 breathlessness

	**Post-COVID-19 breathlessness**		**OR (95% CI)**		
	**No**	**Yes**	**Univariable**	**Multivariable**	**Multiple imputation**
**Sex at birth**					
Male	416 (54.2)	352 (45.8)	1 (ref)	1 (ref)	1 (ref)
Female	195 (42.6)	263 (57.4)	1.59 (1.26–2.01) (p<0.001)	1.44 (1.10–1.90) (p=0.009)	1.56 (1.21–2.00) (p=0.001)
**Age at admission, years**					
<30	10 (40.0)	15 (60.0)	1.12 (0.49–2.63) (p=0.795)	1.20 (0.47–3.12) (p=0.706)	1.35 (0.57–3.23) (p=0.498)
30–39	38 (48.1)	41 (51.9)	0.80 (0.49–1.31) (p=0.378)	0.83 (0.48–1.44) (p=0.511)	0.86 (0.51–1.44) (p=0.568)
40–49	87 (45.8)	103 (54.2)	0.88 (0.62–1.26) (p=0.482)	0.96 (0.64–1.44) (p=0.832)	0.96 (0.66–1.40) (p=0.832)
50–59	151 (42.7)	203 (57.3)	1 (ref)	1 (ref)	1 (ref)
60–69	183 (51.3)	174 (48.7)	0.71 (0.53–0.95) (p=0.022)	0.62 (0.44–0.88) (p=0.007)	0.70 (0.51–0.96) (p=0.025)
70–79	107 (63.7)	61 (36.3)	0.42 (0.29–0.62) (p<0.001)	0.41 (0.26–0.64) (p<0.001)	0.43 (0.28–0.64) (p<0.001)
≥80	29 (72.5)	11 (27.5)	0.28 (0.13–0.57) (p=0.001)	0.27 (0.11–0.60) (p=0.002)	0.31 (0.14–0.66) (p=0.003)
**Index of Multiple Deprivation**					
5, least deprived	127 (55.2)	103 (44.8)	1 (ref)	1 (ref)	1 (ref)
4	111 (51.9)	103 (48.1)	1.14 (0.79–1.66) (p=0.480)	1.30 (0.85–1.99) (p=0.220)	1.22 (0.82–1.81) (p=0.328)
3	116 (50.9)	112 (49.1)	1.19 (0.82–1.72) (p=0.352)	1.21 (0.80–1.84) (p=0.365)	1.22 (0.82–1.79) (p=0.327)
2	135 (50.8)	131 (49.2)	1.20 (0.84–1.71) (p=0.321)	1.31 (0.87–1.96) (p=0.195)	1.20 (0.82–1.76) (p=0.338)
1, most deprived	112 (42.1)	154 (57.9)	1.70 (1.19–2.42) (p=0.004)	1.87 (1.24–2.84) (p=0.003)	1.67 (1.14–2.44) (p=0.009)
**Ethnicity**					
White	422 (48.3)	451 (51.7)	1 (ref)	1 (ref)	1 (ref)
South Asian	82 (53.6)	71 (46.4)	0.81 (0.57–1.14) (p=0.231)	0.83 (0.55–1.26) (p=0.378)	0.80 (0.55–1.17) (p=0.244)
Black	52 (56.5)	40 (43.5)	0.72 (0.46–1.11) (p=0.137)	0.57 (0.34–0.95) (p=0.031)	0.56 (0.35–0.89) (p=0.015)
Mixed	17 (51.5)	16 (48.5)	0.88 (0.44–1.77) (p=0.720)	0.98 (0.44–2.20) (p=0.956)	0.85 (0.41–1.75) (p=0.656)
Other	27 (51.9)	25 (48.1)	0.87 (0.49–1.52) (p=0.616)	0.84 (0.44–1.62) (p=0.606)	0.80 (0.44–1.44) (p=0.448)
**BMI, kg·m^−2^**	31.4±7.1	32.7±7.1	1.03 (1.01–1.04) (p=0.002)	1.08 (0.97–1.21) (p=0.164)	1.08 (0.98–1.19) (p=0.107)
**Number of comorbidities**	2.0±2.0	2.4±2.3	1.09 (1.03–1.15) (p=0.002)	1.09 (1.00–1.18) (p=0.049)	1.08 (1.00–1.17) (p=0.049)
**Pre-existing respiratory condition**					
No	449 (50.3)	444 (49.7)	1 (ref)	1 (ref)	1 (ref)
Yes	162 (48.6)	171 (51.4)	1.07 (0.83–1.37) (p=0.611)	0.85 (0.62–1.17) (p=0.312)	0.82 (0.61–1.11) (p=0.195)
**Pre-existing depression or anxiety**					
No	538 (53.3)	471 (46.7)	1 (ref)	1 (ref)	1 (ref)
Yes	66 (33.3)	132 (66.7)	2.28 (1.66–3.16) (p<0.001)	1.54 (1.00–2.38) (p=0.050)	1.58 (1.06–2.35) (p=0.026)
**Admission duration, days**	13.2±17.2	17.2±22.2	1.01 (1.00–1.02) (p=0.001)	1.01 (1.00–1.02) (p=0.064)	1.01 (1.00–1.02) (p=0.002)
**WHO clinical progression scale**					
WHO class 3–4	110 (49.3)	113 (50.7)	1 (ref)	1 (ref)	1 (ref)
WHO class 5	252 (52.8)	225 (47.2)	0.87 (0.63–1.19) (p=0.388)	0.88 (0.61–1.29) (p=0.522)	0.84 (0.60–1.18) (p=0.314)
WHO class 6	136 (53.1)	120 (46.9)	0.86 (0.60–1.23) (p=0.407)	0.90 (0.58–1.38) (p=0.619)	0.80 (0.54–1.18) (p=0.260)
WHO class 7–9	113 (41.9)	157 (58.1)	1.35 (0.95–1.93) (p=0.097)	1.17 (0.70–1.98) (p=0.548)	0.92 (0.57–1.47) (p=0.715)

Data are presented as n (%) or mean±sd, unless otherwise stated. The dependent variable was post-COVID-19 breathlessness. World Health Organization (WHO) clinical progression scale: not requiring continuous supplemental oxygen (levels 3–4); continuous supplemental oxygen only (level 5); continuous positive airway pressure ventilation, bilevel positive airway pressure or high-flow nasal oxygen (level 6); invasive mechanical ventilation, extra-corporeal membrane oxygenation and acute renal replacement therapy (levels 7–9). The logistic regression model also included body mass index (BMI)^2^.

### Multinomial modelling

Of the 615 participants with post-COVID-19 breathlessness, 213 (35%) had mild and 402 (65%) had severe breathlessness. Compared to those with no post-COVID-19 breathlessness, severe post-COVID-19 breathlessness was associated with the three most deprived quintiles, female sex, pre-existing depression/anxiety and admission duration (table 5). Individuals of Black ethnicity and those in older age groups were less likely to report severe post-COVID-19 breathlessness. Mild post-COVID-19 breathlessness was associated with having more comorbidities and longer admission duration.

**TABLE 5:** Multinomial modelling for post-COVID-19 breathlessness

	**Post-COVID-19 breathlessness OR (95% CI)**	
	**Mild**	**Severe**
**Sex at birth**		
Male	1 (ref)	1 (ref)
Female	1.34 (0.95–1.88)	1.67 (1.26–2.22)
**Age at admission, years**		
<30	2.22 (0.78–6.33)	0.95 (0.35–2.62)
30–39	1.23 (0.62–2.43)	0.68 (0.38–1.22)
40–49	1.38 (0.84–2.26)	0.78 (0.51–1.18)
50–59	1 (ref)	1 (ref)
60–69	0.79 (0.51–1.23)	0.64 (0.46–0.91)
70–79	0.44 (0.25–0.80)	0.39 (0.25–0.62)
≥80	0.58 (0.23–1.41)	0.13 (0.04–0.44)
**Index of Multiple Deprivation**		
5, least deprived	1 (ref)	1 (ref)
4	1.14 (0.68–1.90)	1.26 (0.79–2.00)
3	1.05 (0.63–1.74)	1.38 (0.88–2.18)
2	0.82 (0.49–1.36)	1.52 (0.99–2.35)
1, most deprived	1.02 (0.61–1.71)	2.22 (1.44–3.44)
**Ethnicity**		
White	1 (ref)	1 (ref)
South Asian	0.80 (0.49–1.33)	0.74 (0.48–1.14)
Black	1.02 (0.61–1.71)	0.46 (0.27–0.80)
Mixed	0.76 (0.27–2.15)	0.88 (0.39–1.98)
Other	0.88 (0.39–1.96)	0.77 (0.39–1.49)
**BMI, kg·m^−2^**	0.99 (0.96–1.02)	1.01 (0.99–1.04)
**Number of comorbidities**	1.12 (1.02–1.24)	1.06 (0.98–1.16)
**Pre-existing respiratory condition**		
No	1 (ref)	1 (ref)
Yes	0.94 (0.64–1.39)	0.76 (0.55–1.07)
**Pre-existing depression or anxiety**		
No	1 (ref)	1 (ref)
Yes	1.41 (0.83–2.38)	1.64 (1.06–2.54)
**Admission duration, days**	1.01 (1.00–1.02)	1.02 (1.01–1.02)
**WHO clinical progression scale**		
WHO class 3–4	1 (ref)	1 (ref)
WHO class 5	0.92 (0.59–1.45)	0.81 (0.55–1.20)
WHO class 6	0.76 (0.45–1.31)	0.83 (0.53–1.30)
WHO class 7–9	0.90 (0.48–1.69)	0.98 (0.58–1.65)

The dependent variable was post-COVID-19 breathlessness categorised into three levels, No/Mild/Severe. The reference group (not shown) were those with no post-COVID-19 breathlessness (n=611). Mild post-COVID-19 breathlessness: n=213; severe post-COVID-19 breathlessness: n=402. World Health Organization (WHO) clinical progression scale: not requiring continuous supplemental oxygen (levels 3–4); continuous supplemental oxygen only (level 5); continuous positive airway pressure ventilation, bilevel positive airway pressure or high-flow nasal oxygen (level 6); invasive mechanical ventilation, extra-corporeal membrane oxygenation and acute renal replacement therapy (levels 7–9). BMI: body mass index.

### Prediction model

The multivariable model (equation S1) had fair discriminative ability (C-statistic 0.66, 95% CI 0.63–0.69) (supplementary figure S4) and good calibration (calibration slope 1.00, 95% CI 0.80–1.21; E/O 1.00) despite some under- and over-prediction at higher probabilities (supplementary figure S5).

### Clinical characteristics from the research visit

The period between discharge and research visit was a median 4.7 months (interquartile range 3.4–6.0 months). Fewer participants reviewed between 6 and 8 months after discharge were treated with steroids or antibiotics, and had on average a longer admission duration, and required the highest level of respiratory support compared to individuals who attended a research visit within 6 months of hospitalisation (supplementary tables S4–S6 and figure S6). Despite these differences, the period between discharge and research visit was not associated with post-COVID-19 breathlessness (median 4.7 *versus* 4.7 months; OR 1.00, 95% CI 1.00–1.00) (table 3).

At the research visit, individuals with post-COVID-19 breathlessness had higher scores on the PHQ-9 (median 8.0 *versus* 3.0), GAD-7 (median 5.0 *versus* 2.0) and PCL-5 (median 15.0 *versus* 6.0) than participants without post-COVID-19 breathlessness. With differences in age, sex, ethnicity and socioeconomic status accounted for, individuals with post-COVID-19 breathlessness walked shorter ISWT distances (median 350 *versus* 440 m) with greater leg fatigue afterward (median 3.0 *versus* 2.0) but no difference in oxygen saturations (median 96.0% *versus* 96.0%).

748 (61%) participants completed spirometry at the research visit, with gas transfer available for up to 276 (23%) people. More individuals with post-COVID-19 breathlessness had an FVC less than the LLN (28.0% *versus* 13.8%) and an FEV_1_ less than the LLN (21.5% *versus* 14.6%). However, there was little difference in the presence of obstructive lung function (based on the LLN of FEV_1_/FVC) between those with and without post-COVID-19 breathlessness (3.9% *versus* 6.9%). *K*_CO_ was lower in those with post-COVID-19 breathlessness compared to those without (median 99.6% *versus* 103.5% predicted), while little difference was observed for *T*_LCO_ (median 90.7% *versus* 90.1% predicted).

The following measures from the research visit were associated with increased risk of post-COVID-19 breathlessness (table 3): higher total scores on the PHQ-9 (adjusted OR 1.09, 95% CI 1.07–1.12), GAD-7 (adjusted OR 1.07, 95% CI 1.05–1.09) and PCL-5 (adjusted OR 1.03, 95% CI 1.02–1.04), lower haemoglobin level (adjusted OR 0.88, 95% CI 0.79–0.98 per 1 g·dL^−1^), shorter ISWT distance (adjusted OR 0.91, 95% CI 0.85–0.97 per 100 m), more leg fatigue (adjusted OR 1.14, 95% CI 1.06–1.23), and lower FVC % predicted (adjusted OR 0.98, 95% CI 0.97–0.99).

### Sensitivity analyses

Results from the sensitivity analyses were consistent with results from the primary outcome and are described in the supplementary material (supplementary tables S7–S13 and figure S7).

## Discussion

In this national cohort of 1226 patients who required hospitalisation for COVID-19, half considered their breathlessness to be new or worsening at the research visit compared to before they had COVID-19. Post-COVID-19 breathlessness was associated with the most deprived quintile, pre-existing depression or anxiety, female sex and longer admission duration. Individuals of Black ethnicity and those aged ≥60 years were less likely to report post-COVID-19 breathlessness at follow-up. There was no association between severity of acute COVID-19 and post-COVID-19 breathlessness. At the research visit, participants reporting post-COVID-19 breathlessness had, on average, worse mental health status, lower haemoglobin levels, and walked shorter distances during and had greater leg fatigue after the ISWT. Individuals with post-COVID-19 breathlessness were more likely to have an FEV_1_ and FVC below the LLN compared to those with no change or improvement in breathlessness. However, there was no clear association with *T*_LCO_ or airflow obstruction. Sensitivity analyses supported the primary findings.

Our results have similarities with a French cohort of 478 adults evaluated 3 months after hospitalisation for COVID-19, who found that participants with new or worsening dyspnoea were, on average, younger, and had a longer hospital admission and little or no difference in pulmonary function tests compared to those without new/worsening dyspnoea [18]. In addition, and in keeping with our findings, having a pre-existing respiratory condition was not associated with post-COVID-19 breathlessness [18], which may be explained by individuals with chronic lung disease being used to a background level of breathlessness, which was not considered worse following COVID-19.

In contrast to our study, Jutant *et al.* [18] found that individuals with new/worsening dyspnoea were more likely to have required ITU treatment and have a pulmonary embolism during the admission. Participants in the study by Jutant *et al*. [18] had similarities to participants in our cohort, in respect to median age (61 years), sex (58% male) and the proportion who were diagnosed with pulmonary embolism (9.1%). However, a much greater proportion were intubated (51%), compared to 22% of patients in our study. Prior to COVID-19, Herridge *et al.* [31] found that patients (median age 44 years, 41% without comorbidities) admitted to the ITU with acute respiratory distress syndrome were likely to have ongoing limitations in exercise capacity due to ventilator-induced lung injury, skeletal muscle wasting and deconditioning. Therefore, it might be anticipated that severity of COVID-19 be associated with post-COVID-19 breathlessness. Our cohort was older and with more comorbidities than the sample studied by Herridge *et al.* [31] and fewer participants were intubated than the participants reported by Jutant *et al.* [18]. Therefore, a possible explanation for the lack of association observed between severity of acute COVID-19 and post-COVID-19 breathlessness in our study may be that those not admitted to the ITU had poor pre-morbid health and were more liable to suffer from acute deconditioning than those admitted to the ITU.

Our analyses suggest that post-COVID-19 breathlessness was not associated with objective measures of airflow obstruction and, therefore, less likely to be a consequence of new airway disease. Similarly, we did not see an excess of restrictive patterns in those with post-COVID-19 breathlessness. Individuals with post-COVID-19 breathlessness had, on average, a lower FVC, which may suggest an element of interstitial disease. In the smaller number of individuals who underwent gas transfer tests, *K*_CO_ % predicted was lower in those with post-COVID-19 breathlessness compared to those without, but when adjusted for age, sex, ethnicity and IMD, the association with post-COVID-19 breathlessness was not statistically significant (*i.e.* the confidence intervals overlapped with the null value), making the possibility of fibrosis difficult to confirm. We consider it likely that several factors may have contributed to this observation. Firstly, pulmonary vascular involvement (*e.g.* pulmonary embolism and its sequelae) can contribute to ongoing breathless after acute COVID-19 in the absence of an ongoing clot burden [32]. One possibility is that some individuals with post-COVID-19 breathlessness had subclinical pulmonary emboli during admission [32]. The possible influence of selection bias should also be recognised. Although we aimed to have all patients undertaking all procedures as per protocol, access to more complex lung function tests such as gas transfer was limited and may have resulted in those with clinical features suggesting an interstitial process being more likely to have undergone these tests.

Post-COVID-19 breathlessness was more likely in the most deprived socioeconomic group. Physical activity levels are known to be lowest in the most deprived groups [33], so deprivation may have led to a low exercise tolerance phenotype that was compounded by acute and chronic sequelae of COVID-19. Obesity is also associated with deprivation, as well as chronic breathlessness [16, 34]. Whilst obesity was not associated with post-COVID-19 breathlessness, the mean BMI of this sample was 32.0 kg·m^−2^, which may be an additional contributing factor to the experience of breathlessness. We speculate that post-COVID-19 breathlessness is likely to be a multifactorial and, therefore, heterogeneous problem, which may consist of a decrement in lung function in combination with anxiety or depression, deconditioning, poor exercise tolerance, fatigue and lower haemoglobin. Post-COVID-19 breathlessness may also be influenced by central nervous system perception [35] and whilst we did not collect data specifically to confirm or refute this hypothesis, Jutant *et al.* [18] found that a greater proportion of individuals reporting new or worsening breathlessness scored highly on the Nijmegen questionnaire [36], suggesting a component of dysfunctional breathing.

Regarding interventions for post-COVID-19 breathlessness, our findings suggest that screening for and addressing both the physical and emotional components of breathlessness are likely to be important. In a randomised controlled trial of a 6-week online breathing and wellbeing programme, Philip *et al*. [37] demonstrated improvements in mental health and aspects of breathlessness in people with ongoing symptoms after COVID-19. Interestingly, the intervention led to improvements in the affective, rather than the physical component of the Dyspnoea-12 score, which may suggest that changes in breathlessness experience were related to the emotional impact of the wellbeing programme. Other rehabilitation programmes, which have tended to focus on physical conditioning, have also been shown to improve breathlessness [35, 38], walking distance, lower limb strength and health-related quality of life in patients with persisting symptoms after COVID-19 [35, 38, 39], though corroboration of these results in larger trials would be valuable.

PHOSP-COVID is one of the largest cohorts of post-hospitalisation COVID-19 survivors in the world with comprehensive assessment of participants providing information on physical, psychological, and biochemical characteristics and exposures [6, 7]. This analysis included participants discharged between March 2020 and 31 March 2021, meaning patients treated in hospital both before and after changes in clinical practice for COVID-19 patients (*e.g.* the use of oral steroids [40] or proning during mechanical ventilation [41]) were represented. Limitations include the lack of viral genomic sequencing, vaccination and lung imaging data, which meant that we could not account for vaccination status, radiological abnormalities [18, 19] or the influence that infection with different genetic strains of SARS-CoV-2 may have on post-COVID-19 breathlessness [42]. Participants in this study represent a small proportion of the total number of patients discharged from hospital after treatment for COVID-19 in the UK, which may affect the generalisability of the results. Furthermore, participants in this study were younger than in another, larger sample of hospitalised COVID-19 patients [43], and only included individuals able to attend the research visit. Predicting the influence of this potential selection bias is challenging, because while more severely affected individuals may be underrepresented, it is conceivable that those with ongoing symptoms may have been more willing to participate.

We chose to use patient-reported breathlessness from the PSQ as the primary outcome because it provided a measure of breathlessness both before and after admission for COVID-19. We wanted to account for pre-existing breathlessness in our analyses because being able to identify participants whose breathlessness was new or worsening after COVID-19 was most important to inform policymakers and health services. We acknowledge that as the PSQ breathlessness score before COVID-19 was recorded at the research visit, patient responses may be considered subjective and liable to recall bias. Nevertheless, as the sensitivity analyses supported the associations identified with the primary outcome, we feel that recall bias or subjectivity related to the PSQ breathlessness score has not unduly influenced the main findings. The model derived in this analysis has the potential to predict the probability that an individual discharged following treatment for COVID-19 will experience post-COVID-19 breathlessness. However, a limitation of our work is the lack of model validation, which should be addressed before the prediction model is used.

The multicentre nature of the study and workload pressures on sites meant the period between discharge and follow-up varied. The heterogeneity introduced by considering patient-reported breathlessness from different periods is likely to influence how individuals reported breathlessness, with those reviewed later since hospital discharge having longer time to recover. Compared to individuals who attended a research visit within 6 months of discharge, a higher proportion of participants attending the research visit ≥6 months after discharge had a longer admission duration and required higher levels of respiratory support. A possible explanation for this observation is that the majority of participants attending 6–8 months after hospitalisation were discharged before July 2020 (supplementary figure S6), and were therefore treated earlier in the pandemic and before the use of oral steroids was widespread [40]. However, overall, there was no difference in the period between discharge and the research visit between those reporting post-COVID-19 breathlessness and those not.

In conclusion, post-COVID-19 breathlessness was common in this national cohort of patients hospitalised for COVID-19. Our analysis indicates that individuals discharged following COVID-19 who are from deprived backgrounds, females, <70 years of age, with pre-existing depression or anxiety and who had an admission of over a week, are at greatest risk of new or worsening breathlessness post-COVID-19.

## Supplementary material

10.1183/23120541.00274-2022.Supp1 **Please note:** supplementary material is not edited by the Editorial Office, and is uploaded as it has been supplied by the author.
Supplementary material 00274-2022.SUPPLEMENT
