# Supplementary material for: Characteristics and risk factors for post-COVID-19 breathlessness after hospitalisation for COVID-19
Source: ERJ Open Res. 2023 Feb 20;9(1):00274-2022. doi: 10.1183/23120541.00274-2022 (PMC9790090; doi:10.1183/23120541.00274-2022)
Supplement: Supplementary file 1 [file 00274-2022.SUPPLEMENT.pdf]

Characteristics and risk factors for post-COVID breathlessness following hospitalisation for COVID-19: Supplementary Material

Contents

Details of the PHOSP-COVID Collaborative Group....3

PHOSP-COVID Collaborative Group....3

Supplementary material....13

Subgroup analyses: discharge to review period....23

Sensitivity analyses: PSQ breathlessness at the research visit as a linear outcome....32

Sensitivity analyses: PSQ breathlessness at the research visit as a binary outcome....33

Sensitivity analyses: PSQ breathlessness at the research visit assessed in a multinomial model....39

Sensitivity analyses: Dyspnoea 12 score at the research visit as a linear outcome....40

Reporting guidelines....42

References....45

Figures

Figure S1 Patient Symptom Questionnaire used to collect data on participant breathlessness in the PHOSP-COVID study....13

Figure S2 Breathlessness before COVID-19 and at the research visit stratified by sex, respiratory condition, depression/anxiety, and level of respiratory support received....18

Figure S3 Breathlessness before COVID-19 and at the research visit stratified by age at admission, deprivation, and ethnicity....19

Figure S4 Receiver Operating Characteristic curve for post-COVID breathlessness logistic regression model....21

Figure S5 Calibration plot for post-COVID breathlessness logistic regression model based on the first imputed dataset....22

Figure S6: Discharge date of study participants stratified by the period between discharge and research visit....31

Figure S7 Relationship of PSQ breathlessness and Dyspnoea 12 score collected at the research visit....40

Tables

Table S1 Level of respiratory support received during hospitalization based on the World Health Organization clinical progression scale []....14

Table S2 Thresholds used to determine no, mild and severe post-COVID breathlessness....14

Table S3 Characteristics of included and excluded participants....15

Table S4: Patient characteristics (stratified by discharge to review period)....23

Table S5: Patient characteristics available during hospital admission (stratified by discharge to review period)....26

Table S6: Patient characteristics available at the research visit (stratified by discharge to review period)....28

Table S7 Linear regression model using breathlessness at research visit (PSQ) as the dependent variable....32

Table S8 Patient characteristics stratified by breathlessness at the research visit (PSQ)....33

Table S9 Patient characteristics available during hospital admission, stratified by breathlessness at the research visit (PSQ)....34

Table S10 Patient characteristics available at the research visit, stratified by breathlessness at the research visit (PSQ)....35

Table S11 Multivariable logistic regression with breathlessness at research visit (PSQ) as the dependent variable ..37

Table S12 Multinomial logistic regression for PSQ breathlessness at the research visit....39

Table S13 Linear regression model - Dyspnoea 12 at the research visit....41

Table S14 STROBE Statement—Checklist of items that should be included in reports of cohort studies [2]....42

Table S15 TRIPOD reporting guidelines checklist [3]....43

Equations

Equation S1 Equation for post-COVID breathlessness logistic regression model 20

Details of the PHOSP-COVID Collaborative Group

Writing Group

Luke Daines, Bang Zheng, Omer Elneima, Ewen Harrison, Nazir I Lone, John R Hurst, Jeremy S Brown, Elizabeth Sapey, James D Chalmers, Jennifer K Quint, Samantha Walker, Paul Pfeffer, Salman Siddiqui, Hamish McAuley, Marco Sereno, Aarti Shikotra, Amisha Singapuri, Annemarie B Docherty, Michael Marks, Mark Toshner, Luke S Howard, Alex Horsley, Gisli Jenkins, Joanna C Porter, Ling-Pei Ho, Betty Raman, Louise V Wain, Christopher E Brightling, Rachael A Evans, Liam G Heaney, Anthony De Soyza, Aziz Sheikh.

PHOSP-COVID Collaborative Group

Core Management Group

Chief Investigator C E Brightling, *Members* R A Evans (Lead Co-I), L V Wain (Lead Co-I), J D Chalmers, V C Harris, L P Ho, A Horsley, M Marks, K Poinasamy, B Raman, A Shikotra, A Singapuri

PHOSP-COVID Study Central Coordinating Team

C E Brightling (Chief Investigator), R A Evans (*Lead Co-I*), L V Wain (*Lead Co-I*), R Dowling, C Edwardson, O Elneima, S Finney, N J Greening, B Hargadon, V Harris, L Houchen,, O C Leavy, H J C McAuley, C Overton, T Plekhanova, R Saunders, M Sereno, A Singapuri, A Shikotra, C Taylor, S Terry, C Tong, B Zhao

Steering Committee

Co-chairs D Lomas, E Sapey, *Institution representatives* C Berry, C E Bolton, N Brunskill, E R Chilvers, R Djukanovic, Y Ellis, D Forton, N French, J George, N A Hanley, N Hart, L McGarvey, N Maskell, H McShane, M Parkes, D Peckham, P Pfeffer, A Sayer, A Sheikh, A A R Thompson, N Williams and core management group representation

Executive Board

Chair C E Brightling, representation from the core management group, each working group and platforms

Platforms

Bioresource

W Greenhalf (*Co-Lead*), M G Semple (*Co-Lead*), M Ashworth, H E Hardwick, M Sereno, R Saunders, A Singapuri, V Shaw, A Shikotra, L V Wain

Data Hub

A B Docherty (*Co-Lead*), E M Harrison (*Co-Lead*), A Sheikh (*Co-Lead*), J K Baillie, C E Brightling, L Daines, R A Evans, S Kerr, O C Leavy, N I Lone, H J C McAuley, R Pius, J Quint, M Richardson, M Sereno, M Thorpe, L V Wain

Imaging Alliance

M Halling-Brown (*Co-Lead*), F Gleeson (*Co-Lead*), J Jacob (*Co-Lead*), S Neubauer (*Co-Lead*) B Raman (*Co-Lead*) S Siddiqui (*Co-Lead*) J M Wild (*Co-Lead*), S Aslani, P Jezzard, H Lamlum, W Lilaonitkul, E Tunnicliffe, J Willoughby

Omics

L V Wain (*Co-Lead)*, J K Baillie (*Co-Lead*), H Baxendale, C E Brightling, M Brown, J D Chalmers, R A Evans, B Gooptu, W Greenhalf, H E Hardwick, R G Jenkins, D Jones, I Koychev, C Langenberg, A Lawrie, P L Molyneaux, A Shikotra, J Pearl, M Ralser, N Sattar, R Saunders, J T Scott, T Shaw, D Thomas, D Wilkinson

Working Groups

Airways

L G Heaney (*Co-Lead*), A De Soyza (*Co-Lead*), D Adeloye, C E Brightling, J S Brown, J Busby, J D Chalmers, C Echevarria, L Daines, O Elneima, RA Evans, J Hurst, P Novotny, P Pfeffer, K Poinasamy, J Quint, I Rudan, E Sapey, A Sheikh, S Siddiqui, S Walker

Brain

J R Geddes (*Lead*), M Hotopf *(Co-Lead)*, K Abel, R Ahmed, L Allan, C Armour, D Baguley, D Baldwin, C Ballard, J Bambrough, K Bhui, G Breen, M Broome, T Brugha, E Bullmore, D Burn, J Cavanagh, T Chalder, D Clark, A David, B Deakin, H Dobson, B Elliott, J Evans, R Francis, E Guthrie, P Harrison, M Henderson, A Hosseini, N Huneke, M Husain, T Jackson, I Jones, T Kabir, P Kitterick, A Korszun, I Koychev, J Kwan, A Lingford-Hughes, C Mackay, P Mansoori, H McAllister-Williams, K McIvor, B Michael, L Milligan, R Morriss, E Mukaetova-Ladinska, T Nicholson, S Paddick, C Pariante, J Pimm, K Saunders, M Sharpe, G Simons, R Upthegrove, S Wessely

Cardiac

G P McCann (*Lead*), C Antoniades, R Bell, A Bularga, C Berry, K M Channon, P Chowienczyk, J P Greenwood, A Hingorani, A D Hughes, C Jolley, K Khunti, C Kotanidis, J Mayet, N L Mills, A J Moss, S Neubauer, D E Newby, B Raman, N Samani, A Saratzis, N Sattar, A M Shah, C L Sudlow, M Toshner, R Touyz, B Williams, C Xie

Immunology

P J M Openshaw (*Lead*), D Altmann, J K Baillie, R Batterham, H Baxendale, N Bishop, C E Brightling, P C Calder, R A Evans, J L Heeney, T Hussell, P Klenerman, J M Lord, P Moss, S L Rowland-Jones, W Schwaeble, M G Semple, R S Thwaites, L Turtle, L V Wain, S Walmsley, D Wraith

Intensive Care

M J Rowland (*Lead*), A Rostron (*Co-Lead*), J K Baillie, B Connolly, A B Docherty, N I Lone, D F McAuley, D Parekh, J Simpson, C Summers

Lung Fibrosis

R G Jenkins (*Co-Lead*), J Porter (*Co-Lead*), R J Allen, R Aul, J K Baillie, S Barratt, P Beirne, J Blaikley, R C Chambers, N Chaudhuri, C Coleman, L Fabbri, P M George, M Gibbons, F Gleeson, B Gooptu, I Hall, N A Hanley, L P Ho, E Hufton, J Jacob, S Johnson, M Jones, S Jones, F Khan, J Mitchell, P L Molyneaux, J E Pearl, K Piper Hanley, K Poinasamy, J Quint, P Rivera-Ortega, M G Semple, J Simpson, M Spears, L G Spencer, S Stanel, I Stewart, D Thickett, R Thwaites, L V Wain, S Walker, S Walsh, J M Wild, D G Wootton, L Wright

Metabolic

S Heller (*Co-Lead*), M Davies (*Co-Lead*), H Atkins, S Bain, J Dennis, K Ismail, D Johnston, P Kar, K Khunti, C Langenberg, P McArdle, A McGovern, T Peto, J Petrie, E Robertson, N Sattar, K Shah, J Valabhji, B Young

Pulmonary and Systematic Vasculature

L Howard (*Co-Lead*), Mark Toshner (*Co-Lead*), C Berry, P Chowienczyk, D Lasserson, A Lawrie, O C Leavy, J Mitchell, L Price, J Quint, J Rossdale, N Sattar, C Sudlow, A A R Thompson, J M Wild, M Wilkins

Rehabilitation, Sarcopenia and Fatigue

S J Singh (*Co-Lead*), W D-C Man (*Co-Lead*), J M Lord (*Co-Lead*), N J Greening (*Co-Lead*), T Chalder (*Co-Lead*), J T Scott (*Co-Lead*), N Armstrong, E Baldry, N Basu, M Beadsworth, L Bishop, C E Bolton, A Briggs, M Buch, G Carson, J Cavanagh, H Chinoy, E Daynes, S Defres, R A Evans, P Greenhaff, S Greenwood, M Harvie, M Husain, A McArdle, A McMahon, M McNarry, C Nolan, J Pimm, J Sargent, L Sigfrid, M Steiner, D Stensel, A L Tan, J Whitney, D Wilkinson, D Wilson, M Witham, D G Wootton, T Yates

Renal

D Thomas (*Lead*), N Brunskill (*Co-Lead*), S Francis (*Co-Lead*), S Greenwood (*Co-Lead*), C Laing (*Co-Lead*), K Bramham, P Chowdhury, A Frankel, L Lightstone, S McAdoo, K McCafferty, M Ostermann, N Selby, C Sharpe, M Willicombe

Local Clinical Centre PHOSP-COVID trial staff

(listed in alphabetical order)

Airedale NHS Foundation Trust

A Shaw (PI), L Armstrong, B Hairsine, H Henson, C Kurasz, L Shenton

Aneurin Bevan University Health Board

S Fairbairn (PI), A Dell, N Hawkings, J Haworth, M Hoare, A Lucey, V Lewis, G Mallison, H Nassa, C Pennington, A Price, C Price, A Storrie, G Willis, S Young

Barts Health NHS Trust & Queen Mary University of London

P Pfeffer (PI), K Chong-James, C David, W Y James, A Martineau, O Zongo

Barnsley Hospital NHS Foundation Trust
A Sanderson (PI)

Belfast Health and Social Care Trust & Queen's University Belfast
L G Heaney (PI), C Armour, V Brown, T Craig, S Drain, B King, N Magee, D McAulay, E Major, L McGarvey, J McGinness, R Stone

Betsi Cadwaladr University Health Board
A Haggar (PI), A Bolger, F Davies, J Lewis, A Lloyd, R Manley, E McIvor, D Menzies, K Roberts, W Saxon, D Southern, C Subbe, V Whitehead

Borders General Hospital, NHS Borders
H El-Taweel (PI), J Dawson, L Robinson

Bradford Teaching Hospitals NHS Foundation Trust
D Saralaya (PI), L Brear, K Regan, K Storton

Cambridge University Hospitals NHS Foundation Trust, NIHR Cambridge Clinical Research Facility & University of Cambridge
J Fuld (PI), A Bermperi, I Cruz, K Dempsey, A Elmer, H Jones, S Jose, S Marciniak, M Parkes, C Ribeiro, J Taylor, M Toshner, L Watson, J Worsley

Cardiff and Vale University Health Board
R Sabit (PI), L Broad, A Buttress, T Evans, M Haynes, L Jones, L Knibbs, A McQueen, C Oliver, K Paradowski, J Williams

Chesterfield Royal Hospital NHS Trust
E Harris (PI), C Sampson

Cwm Taf Morgannwg University Health Board
C Lynch (PI), E Davies, C Evenden , A Hancock, K Hancock, M Rees , L Roche, N Stroud, T Thomas-Woods

East Cheshire NHS Trust
M Babores (PI), J Bradley-Potts, M Holland, N Keenan, S Shashaa, H Wassall

East Kent Hospitals University NHS Foundation Trust
E Beranova (PI), H Weston (PI), T Cosier, L Austin, J Deery, T Hazelton, C Price, H Ramos, R Solly, S Turney

Gateshead NHS Trust

L Pearce (PI), W McCormack, S Pugmire, W Stoker, A Wilson

Guy's and St Thomas' NHS Foundation Trust
N Hart (PI), LA Aguilar Jimenez, G Arbane, S Betts, K Bisnauthsing, A Dewar, P Chowdhury, A Dewar, G Kaltsakas, H Kerslake, MM Magtoto, P Marino, LM Martinez, M Ostermann, J Rossdale, TS Solano, E Wynn

Hampshire Hospitals NHS Foundation Trust
N Williams (PI), W Storrar (PI), M Alvarez Corral, A Arias, E Bevan, D Griffin, J Martin, J Owen, S Payne, A Prabhu, A Reed, C Wrey Brown

Harrogate and District NHD Foundation Trust

C Lawson (PI), T Burdett, J Featherstone, A Layton, C Mills, L Stephenson,

Hull University Teaching Hospitals NHS Trust & University of Hull
N Easom (PI), P Atkin, K Brindle, M G Crooks, K Drury, R Flockton, L Holdsworth, A Richards, D L Sykes, S Thackray-Nocera, C Wright

Hywel Dda University Health Board

K E Lewis (PI), A Mohamed (PI), G Ross (PI), S Coetzee, K Davies, R Hughes, R Loosley, L O'Brien, Z Omar, H McGuinness, E Perkins, J Phipps, A Taylor, H Tench, R Wolf-Roberts

Imperial College Healthcare NHS Trust & Imperial College London
L Howard (PI), O Kon (PI), D C Thomas (PI), S Anifowose, L Burden, E Calvelo, B Card, C Carr, E R Chilvers, D Copeland, P Cullinan, P Daly, L Evison, T Fayzan, H Gordon, S Haq, R G Jenkins, C King, K March, M Mariveles, L McLeavey, N Mohamed, S Moriera, U Munawar, J Nunag, U Nwanguma, L Orriss- Dib, A Ross, M Roy, E Russell, K Samuel, J Schronce, N Simpson, L Tarusan, C Wood, N Yasmin

Kettering General Hospital NHS Trust

R Reddy (PI), A-M, Guerdette, M Hewitt, K Warwick, S White

King's College Hospital NHS Foundation Trust & Kings College London
A M Shah (PI), C J Jolley (PI), O Adeyemi, R Adrego, H Assefa-Kebede, J Breeze, M Brown, S Byrne, T Chalder, P Dulawan, N Hart, A Hayday, A Hoare, A Knighton, M Malim, S Patale, I Peralta, N Powell, A Ramos, K Shevket, F Speranza, A Te

Leeds Teaching Hospitals & University of Leeds
P Beirne (PI), A Ashworth, J Clarke, C Coupland, M Dalton, E Wade, C Favager, J Greenwood, J Glossop, L Hall, T Hardy, A Humphries, J Murira, D Peckham, S Plein, J Rangeley, G Saalmink, A L Tan, B Whittam, N Window, J Woods

Lewisham & Greenwich NHS Trust

G Coakley (PI)

Liverpool University Hospitals NHS Foundation Trust & University of Liverpool

D G Wootton (PI), L Turtle (PI), L Allerton, AM All, M Beadsworth, A Berridge, J Brown, S Cooper, A Cross, S Defres, S L Dobson, J Earley, N French, W Greenhalf, H E Hardwick, K Hainey, J Hawkes, V Highett, S Kaprowska, AL Key, L Lavelle-Langham, N Lewis-Burke, G Madzamba, F Malein, S Marsh, C Mears, L Melling, M J Noonan, L Poll, J Pratt, E Richardson, A Rowe, M G Semple, V Shaw, K A Tripp, L O Wajero, S A Williams-Howard, J Wyles,

London North West University Healthcare NHS Trust
S N Diwanji (PI), P Papineni (PI), S Gurram, S Quaid, G F Tiongson, E Watson

Manchester University NHS Foundation Trust & University of Manchester
B Al-Sheklly (PI), A Horsley (PI), C Avram, J Blaikely, M Buch, N Choudhury, D Faluyi, T Felton, T Gorsuch, N A Hanley, T Hussell, Z Kausar, N Odell, R Osbourne, K Piper Hanley, K Radhakrishnan, S Stockdale

Newcastle upon Tyne Hospitals NHS Foundation Trust & University of Newcastle
A De Soyza (PI), C Echevarria (PI), A Ayoub, J Brown, G Burns, G Davies, H Fisher, C Francis, A Greenhalgh, P Hogarth, J Hughes, K Jiwa, G Jones, G MacGowan, D Price, A Sayer, J Simpson, H Tedd, S Thomas, S West, M Witham, S Wright, A Young

NHS Dumfries and Galloway
M J McMahon (PI), P Neill

NHS Greater Glasgow and Clyde Health Board & University of Glasgow
D Anderson (PI), H Bayes (PI), C Berry (PI), D Grieve (PI), I B McInnes (PI), N Basu, A Brown, A Dougherty, K Fallon, L Gilmour, K Mangion, A Morrow, K Scott, R Sykes

NHS Highland
E K Sage (PI), F Barrett, A Donaldson

NHS Lanarkshire
M Patel (PI), D Bell, A Brown, M Brown, R Hamil, K Leitch, L Macliver, J Quigley, A Smith, B Welsh

NHS Lothian & University of Edinburgh

G Choudhury (PI), J K Baillie, S Clohisey, A Deans, A B Docherty, J Furniss, E M Harrison, S Kelly, N I Lone, A Sheikh

NHS Tayside & University of Dundee
J D Chalmers (PI), D Connell, A Elliott, C Deas, J George, S Mohammed, J Rowland, A R Solstice, D Sutherland, C J Tee

North Bristol NHS Trust & University of Bristol

N Maskell (PI), D Arnold, S Barrett, H Adamali, A Dipper, S Dunn, A Morley, L Morrison, L Stadon, S Waterson, H Welch

North Middlesex Hospital NHS Trust
B Jayaraman (PI), T Light

Nottingham University Hospitals NHS Trust & University of Nottingham
C E Bolton (PI), P Almeida, J Bonnington, M Chrystal, P Greenhaff, A Gupta, L Howard, S Linford, L Matthews, R Needham, K Shaw, A K Thomas

Oxford University Hospitals NHS Foundation Trust & University of Oxford
L P Ho (PI), N M Rahman (PI), M Ainsworth, A Alamoudi, A Bates, A Bloss, A Burns, P Carter, J Chen, F Conneh, T Dong, R I Evans, E Fraser, X Fu, J R Geddes, F Gleeson, P Harrison, M Havinden-Williams, P Jezzard, N Kanellakis, I Koychev, P Kurupati, X Li, H McShane, C Megson, K Motohashi, S Neubauer, D Nicoll, G Ogg, E Pacpaco, M Pavlides, Y Peng, N Petousi, N Rahman, B Raman, M J Rowland, K Saunders, M Sharpe, N Talbot, E Tunnicliffe

Royal Brompton and Harefield Clinical Group, Guy's and St Thomas' NHS Foundation Trust.
W Man (PI), B Patel (PI), R E Barker, D Cristiano, N Dormand, M Gummadi, S Kon, K Liyanage, C M Nolan, S Patel, O Polgar, P Shah, S J Singh, J A Walsh

Royal Free London NHS Foundation Trust
J Hurst (PI), H Jarvis (PI), S Mandal (PI), S Ahmad, S Brill, L Lim, D Matila, O Olaosebikan, C Singh

Royal Papworth Hospital NHS Foundation Trust
M Toshner (PI), H Baxendale, L Garner, C Johnson, J Mackie, A Michael, J Pack, K Paques, H Parfrey, J Parmar

Salford Royal NHS Foundation Trust
N Diar Bakerly (PI), P Dark, D Evans, E Hardy, A Harvey, D Holgate, S Knight, N Mairs, N Majeed, L McMorrow, J Oxton, J Pendlebury, C Summersgill, R Ugwuoke, S Whittaker

Salisbury NHS Foundation Trust
W Matimba-Mupaya (PI), S Strong-Sheldrake

Sheffield Teaching NHS Foundation Trust & University of Sheffield
S Rowland-Jones (PI), A A R Thompson (Co PI), J Bagshaw, M Begum, K Birchall, R Butcher, H Carborn, F Chan, K Chapman, Y Cheng, L Chetham, C Clark, Z Coburn, J Cole, M Dixon, A Fairman, J Finnigan, H Foot, D Foote, A Ford, R Gregory, K Harrington, L Haslam, L Hesselden, J Hockridge, A Holbourn, B Holroyd-Hind, L Holt, A Howell, E Hurditch, F Ilyas, C Jarman, A Lawrie, E Lee, J-H Lee, R Lenagh, A Lye, I Macharia, M Marshall, A Mbuyisa, J McNeill, S Megson, J Meiring, L Milner, S Misra, H Newell, T Newman, C Norman, L Nwafor, D Pattenadk, M Plowright, J Porter, P Ravencroft, C Roddis, J Rodger, P Saunders, J Sidebottom, J Smith, L Smith, N Steele, G Stephens, R Stimpson, B Thamu, N Tinker, K Turner, H Turton, P Wade, S Walker, J Watson, I Wilson, A Zawia

St George's University Hospitals NHS Foundation Trust
R Aul (PI), M Ali, A Dunleavy (PI), D Forton, N Msimanga, M Mencias, T Samakomva, S Siddique, J Teixeira, V Tavoukjian

Sherwood Forest Hospitals NHS Foundation Trust
J Hutchinson (PI), L Allsop, K Bennett, P Buckley, M Flynn, M Gill, C Goodwin, M Greatorex, H Gregory, C Heeley, L Holloway, M Holmes, J Kirk, W Lovegrove, TA Sewell, S Shelton, D Sissons, K Slack, S Smith, D Sowter, S Turner, V Whitworth, I Wynter

Shropshire Community Health NHS Trust
L Warburton (PI), S Painter, J Tomlinson

Somerset NHS Foundation Trust
C Vickers (PI), T Wainwright, D Redwood, J Tilley, S Palmer

Swansea Bay University Health Board
G A Davies (PI), L Connor, A Cook, T Rees, F Thaivalappil, C Thomas

Tameside and Glossop Integrated Care NHS Foundation

A Butt (PI), M Coulding, H Jones, S Kilroy, J McCormick, J McIntosh, H Savill, V Turner, J Vere

The Great Western Hospital Foundation Trust

E Fraile (PI), J Ugoji

The Hillingdon Hospitals NHS Foundation Trust
S S Kon (PI), H Lota, G Landers, M Nasseri, S Portukhay

The Rotherham NHS Foundation Trust

A Hormis (PI), A Daniels, J Ingham, L Zeidan

United Lincolnshire Hospitals NHS Trust
M Chablani (PI), L Osborne

University College London Hospital & University College London
M Marks (PI), J S Brown (PI), N Ahwireng, B Bang, D Basire, R C Chambers, A Checkley, R Evans, M Heightman, T Hillman, J Hurst, J Jacob, S Janes, R Jastrub, M Lipman, S Logan, D Lomas, M Merida Morillas, H Plant, J C Porter, K Roy, E Wall

University Hospital Birmingham NHS Foundation Trust & University of Birmingham
D Parekh (PI), N Ahmad Haider, C Atkin, R Baggott, M Bates, A Botkai, A Casey, B Cooper, J Dasgin, K Draxlbauer, N Gautam, J Hazeldine, T Hiwot, S Holden, K Isaacs, T Jackson, S Johnson, V Kamwa, D Lewis, J M Lord, S Madathil, C McGhee, K Mcgee, A Neal, A Newton Cox, J Nyaboko, D Parekh, Z Peterkin, H Qureshi, L Ratcliffe, E Sapey, J Short, T Soulsby, J Stockley, Z Suleiman, T Thompson, M Ventura, S Walder, C Welch, D Wilson, S Yasmin, K P Yip

University Hospitals of Derby and Burton

P Beckett (PI) C Dickens, U Nanda

University Hospitals of Leicester NHS Trust & University of Leicester

C E Brightling (CI), R A Evans (PI), M Aljaroof, N Armstrong, H Arnold, H Aung, M Bakali, M Bakau, M Bingham, M Bourne, C Bourne, N Brunskill, P Cairns, L Carr, A Charalambou, C Christie, M Davies, S Diver, S Edwards, C Edwardson, O Elneima, H Evans, J Finch, S Glover, N Goodman, B Gootpu, N J Greening, K Hadley, P Haldar, B Hargadon, V C Harris, L Houchen, W Ibrahim, L Ingram, K Khunti, A Lea, D Lee, G P McCann, H J C McAuley, P McCourt, T Mcnally, A Moss, W Monteiro, M Pareek, S Parker, A Rowland, A Prickett, I N Qureshi, R Russell, M Sereno, A Shikotra, S Siddiqui, A Singapuri, S J Singh, J Skeemer, M Soares, E Stringer, T Thornton, M Tobin, L V Wain, T J C Ward, F Woodhead, T Yates, A Yousuf

University Hospital Southampton NHS Foundation Trust & University of Southampton
M Jones (PI), C Childs, R Djukanovic, S Fletcher, M Harvey, E Marouzet, B Marshall, R Samuel, T Sass, T Wallis, H Wheeler

Whittington Health NHS
R Dharmagunawardena (PI), E Bright, P Crisp, M Stern

Wirral University Teaching Hospital

A Wight (PI), L Bailey, A Reddington

Wrightington Wigan and Leigh NHS trust

A Ashish (PI), J Cooper, E Robinson

Yeovil District Hospital NHS Foundation Trust

A Broadley (PI)

York & Scarborough NHS Foundation Trust

K Howard (PI), L Barman, C Brookes, K Elliott. L Griffiths, Z Guy, D Ionita, H Redfearn, C Sarginson

A Turnbull

Health and Care Research Wales

Y Ellis

London School of Hygiene & Tropical Medicine (LSHTM)
M Marks, A Briggs

NIHR Office for Clinical Research Infrastructure

K Holmes

Patient Public Involvement Leads

Asthma UK and British Lung Foundation Partnership - K Poinasamy, S Walker

Royal Surrey NHS Foundation Trust
M Halling-Brown

South London and Maudsley NHS Foundation Trust & Kings College London
G Breen, M Hotopf

Swansea University & Swansea Welsh Network

K Lewis, N Williams

Supplementary material

Figure S1 Patient Symptom Questionnaire used to collect data on participant breathlessness in the PHOSP-COVID study

Please complete the following questionnaire

If anything is unclear or there is something you are concerned about, please discuss this with your clinician or nurse during your appointment.

a) Do you feel fully recovered from COVID-19? ☐ **Yes** ☐ **No** ☐ **Not sure**

We would like to understand more about your symptoms. For each symptom below (b to f), Please rate them before you had COVID-19, in general since you had COVID-19 and the worst you have felt in the past 24 hours.

Please rate them on a scale 0-10 where:

← 0 1 2 3 4 5 6 7 8 9 10

NEUTRAL SLIGHT SENSATION ANNOYING DISTRESSING UNBEARABLE

	**Please rate these symptoms on a scale of 0 - 10**			Please also try to indicate if the symptom is staying the same, getting better or getting worse
	i) Before you had COVID-19	ii) Since you had COVID-19	iii) Worst in last 24hrs	iv) Trajectory
**b)** Breathlessness				☐ Same ☐ Better ☐ Worse
**c)** Cough				☐ Same ☐ Better ☐ Worse
**d)** Fatigue				☐ Same ☐ Better ☐ Worse
**e)** Sleep quality				☐ Same ☐ Better ☐ Worse
**f)** Pain				☐ Same ☐ Better ☐ Worse

Table S1 Level of respiratory support received during hospitalization based on the World Health Organization clinical progression scale [1]

Levels	Definition
3-4	Not requiring continuous supplemental oxygen
5	Continuous supplemental oxygen only
6	Continuous Positive Airway Pressure ventilation, Bi-Level Positive Airway Pressure or High Flow Nasal Oxygen.
7-9	Invasive Mechanical Ventilation, Extra-Corporeal Membrane Oxygenation and acute Renal Replacement Therapy.

Table S2 Thresholds used to determine no, mild and severe post-COVID breathlessness

To investigate potential differences between individuals according to the severity of post-COVID breathlessness, multinomial modelling was used. Using the change in PSQ breathlessness score at the time of the research visit compared to before COVID-19, we created a categorical (ordinal) variable using the following thresholds:

Post-COVID breathlessness: Levels	Definition
No	Change in PSQ ≤ 0
Yes: Mild	Change in PSQ = 1 or 2.
Yes: Severe	Change in PSQ ≥ 3

Table S3 Characteristics of included and excluded participants

Of the 1,843 individuals who completed a research visit between 1 and 8 months, 617 had missing data for breathlessness before COVID-19 or breathlessness at the first research assessment and were therefore excluded from this analysis.

	Total N	Levels	Eligible for inclusion		Total
			No	Yes	
Total N (%)			617 (33.5)	1226 (66.5)	1843
Age at admission (years)	1824 (99.0)	50-59	157 (25.4)	354 (28.9)	511 (27.7)
		<30	21 (3.4)	25 (2.0)	46 (2.5)
		30-39	36 (5.8)	79 (6.4)	115 (6.2)
		40-49	87 (14.1)	190 (15.5)	277 (15.0)
		60-69	185 (30.0)	357 (29.1)	542 (29.4)
		70-79	108 (17.5)	168 (13.7)	276 (15.0)
		80+	17 (2.8)	40 (3.3)	57 (3.1)
		(Missing)	6 (1.0)	13 (1.1)	19 (1.0)
Sex at birth	1843 (100.0)	Male	372 (60.3)	768 (62.6)	1140 (61.9)
		Female	245 (39.7)	458 (37.4)	703 (38.1)
Ethnicity	1800 (97.7)	White	440 (71.3)	873 (71.2)	1313 (71.2)
		South Asian	74 (12.0)	153 (12.5)	227 (12.3)
		Black	47 (7.6)	92 (7.5)	139 (7.5)
		Mixed	9 (1.5)	33 (2.7)	42 (2.3)
		Other	27 (4.4)	52 (4.2)	79 (4.3)
		(Missing)	20 (3.2)	23 (1.9)	43 (2.3)
Index of multiple deprivation	1811 (98.3)	1 - most deprived	120 (19.4)	266 (21.7)	386 (20.9)
		2	165 (26.7)	266 (21.7)	431 (23.4)
		3	99 (16.0)	228 (18.6)	327 (17.7)
		4	105 (17.0)	214 (17.5)	319 (17.3)
		5 - least deprived	118 (19.1)	230 (18.8)	348 (18.9)

	Total N	Levels	Eligible for inclusion		Total
			No	Yes	
		(Missing)	10 (1.6)	22 (1.8)	32 (1.7)
BMI	1509 (81.9)	Mean (SD)	32.1 (7.1)	32.0 (7.1)	32.1 (7.1)
Smoking	1622 (88.0)	Never	217 (35.2)	689 (56.2)	906 (49.2)
		Ex-smoker	181 (29.3)	506 (41.3)	687 (37.3)
		Current smoker	11 (1.8)	18 (1.5)	29 (1.6)
		(Missing)	208 (33.7)	13 (1.1)	221 (12.0)
Number of comorbidities	1843 (100.0)	Median (IQR)	2.0 (1.0 to 3.0)	2.0 (0.0 to 3.0)	2.0 (1.0 to 3.0)
Cardiovascular	1843 (100.0)	No	310 (50.2)	679 (55.4)	989 (53.7)
		Yes	307 (49.8)	547 (44.6)	854 (46.3)
Respiratory	1843 (100.0)	No	442 (71.6)	893 (72.8)	1335 (72.4)
		Yes	175 (28.4)	333 (27.2)	508 (27.6)
Depression or anxiety	1821 (98.8)	No	498 (80.7)	1009 (82.3)	1507 (81.8)
		Yes	116 (18.8)	198 (16.2)	314 (17.0)
During hospital admission					
Admission duration (days)	1841 (99.9)	Median (IQR)	7.0 (4.0 to 14.0)	8.0 (4.0 to 17.0)	8.0 (4.0 to 16.0)
WHO clinical progression scale	1843 (100.0)	WHO – class 3-4	93 (15.1)	223 (18.2)	316 (17.1)
		WHO – class 5	276 (44.7)	477 (38.9)	753 (40.9)
		WHO – class 6	155 (25.1)	256 (20.9)	411 (22.3)
		WHO – class 7-9	93 (15.1)	270 (22.0)	363 (19.7)
Proning during mechanical ventilation	1683 (91.3)	No	485 (78.6)	892 (72.8)	1377 (74.7)
		Yes	96 (15.6)	210 (17.1)	306 (16.6)
		(Missing)	36 (5.8)	124 (10.1)	160 (8.7)
Pulmonary Embolism	1737 (94.2)	No	534 (86.5)	1025 (83.6)	1559 (84.6)
		Yes	57 (9.2)	121 (9.9)	178 (9.7)
		(Missing)	26 (4.2)	80 (6.5)	106 (5.8)
Coronary thrombosis	1729 (93.8)	No	587 (95.1)	1135 (92.6)	1722 (93.4)

	Total N	Levels	Eligible for inclusion		Total
			No	Yes	
		Yes	<5 (-)	<5 (-)	7 (0.4)
		(Missing)	- (-)	- (-)	114 (6.2)
Antibiotic therapy	1789 (97.1)	No	118 (19.1)	236 (19.2)	354 (19.2)
		Yes	484 (78.4)	951 (77.6)	1435 (77.9)
		(Missing)	15 (2.4)	39 (3.2)	54 (2.9)
Systemic steroids (Oral or IV)	1738 (94.3)	No	258 (41.8)	613 (50.0)	871 (47.3)
		Yes	336 (54.5)	531 (43.3)	867 (47.0)
		(Missing)	23 (3.7)	82 (6.7)	105 (5.7)
Therapeutic dose anti-coagulation	1740 (94.4)	No	344 (55.8)	685 (55.9)	1029 (55.8)
		Yes	246 (39.9)	465 (37.9)	711 (38.6)
		(Missing)	27 (4.4)	76 (6.2)	103 (5.6)

Figure S2 Breathlessness before COVID-19 and at the research visit stratified by sex, respiratory condition, depression/anxiety, and level of respiratory support received

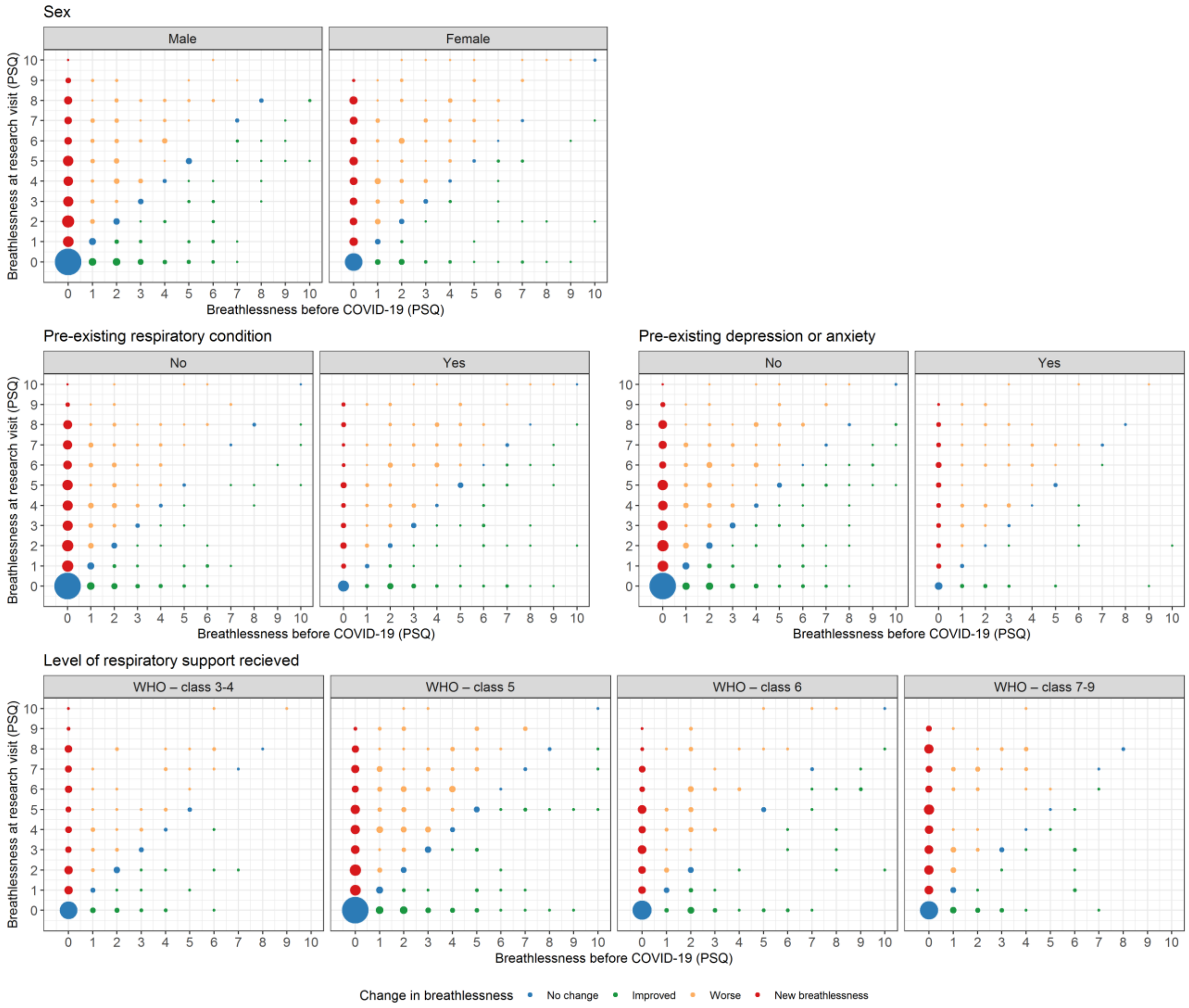

Figure S3 Breathlessness before COVID-19 and at the research visit stratified by age at admission, deprivation, and ethnicity

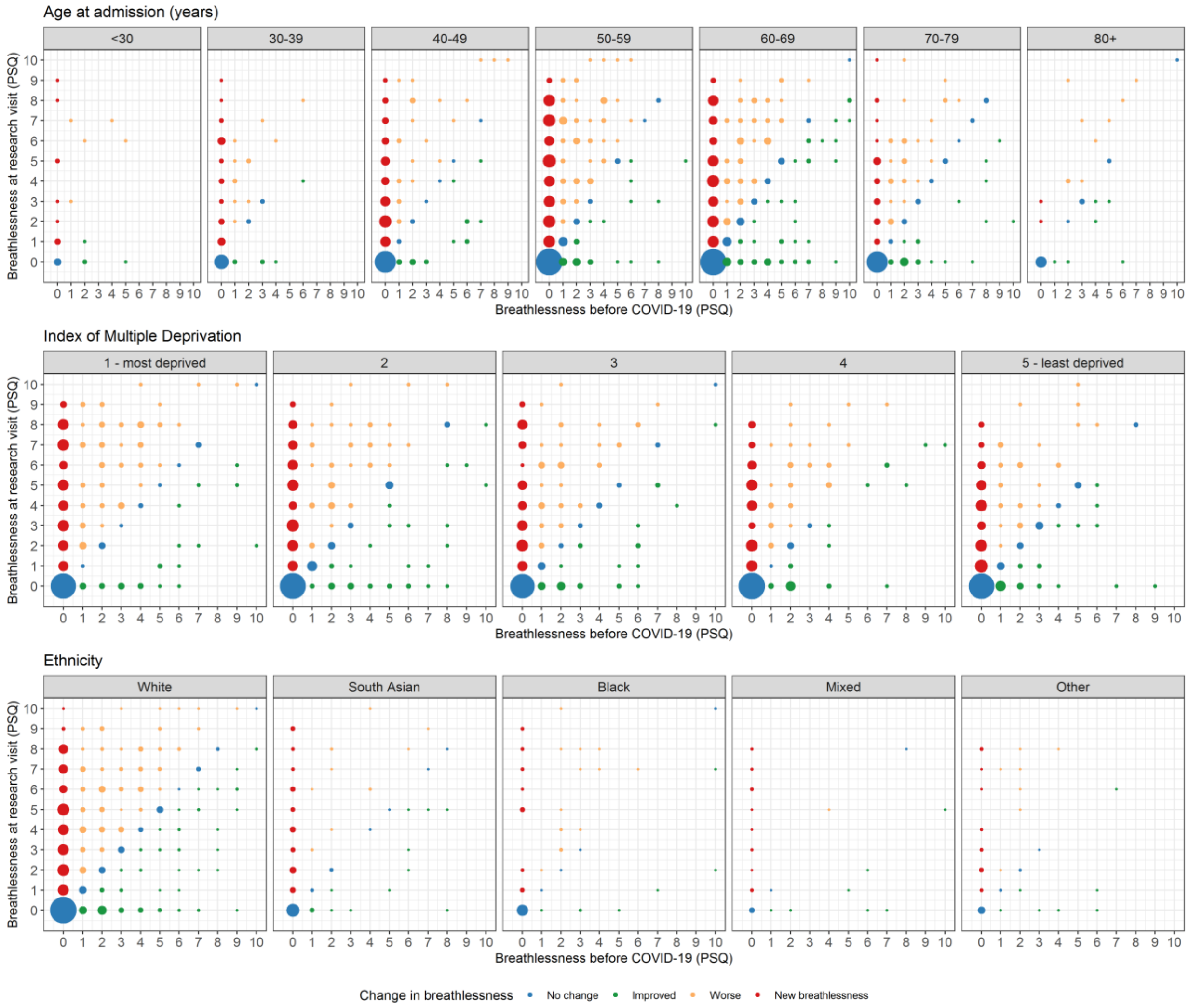

Equation S1 Equation for post-COVID breathlessness logistic regression model

$$\ln\left(\frac{p_{(post-COVID\ breathlesness)}}{1-p_{(post-COVID\ breathlessness)}}\right) = -1.75 + 0.44(Female) + 0.30(age < 30) - 0.15(age\ 30\ to\ 39) - 0.04(age\ 40\ to\ 49) - 0.36(age\ 50\ to\ 59)$$

$$- 0.85(age\ 70\ to\ 79) - 1.18(age\ \geq 80) + 0.20(IMD\ 4) + 0.19(IMD\ 3) + 0.18(IMD\ 2) + 0.51(IMD\ 1) - 0.22(Ethnicity\ South\ Asian)$$

$$- 0.59(Ethnicity\ Black) - 0.16(Ethnicity\ Mixed) - 0.23(Ethnicity\ Other) + 0.08(BMI) + 0.08(Number\ of\ comorbidities)$$

$$- 0.20(existing\ respiratory\ disease) + 0.46(existing\ depression\ or\ anxiety) + 0.01(admission\ duration) - 0.18(WHO\ class\ 5)$$

$$- 0.23(WHO\ class\ 6) - 0.09(WHO\ Class\ 7\ to\ 9)$$

Notes:

- Age should be inputted as years
- Ethnicity:
  - recorded as “Black” if participants selected: Black, African, Caribbean, Black British
  - recorded as “South Asian” if participants selected Indian, Pakistani, Bangladeshi, Other Asian background.
  - recorded Mixed if participants selected White and Black African, White and Asian, White and Black Caribbean, any other Mixed/Multiple ethnic background.
  - recorded as “Other” if participants selected Arab, Chinese, any other ethnic group
- Number of co-morbidities should be inputted as an integer ≥ 0.
- BMI = Body Mass Index.
- Admission duration should be inputted in days.
- World Health Organization (WHO) clinical progression scale:
  - continuous supplemental oxygen only (level 5);
  - Continuous Positive Airway Pressure (CPAP) ventilation, Bi-Level Positive Airway Pressure or High Flow Nasal Oxygen (HFNO) (level 6);
  - Invasive Mechanical Ventilation (IMV), Extra-Corporeal Membrane Oxygenation (ECMO) and acute Renal Replacement Therapy (RRT) (levels 7-9).
- The logistic regression model also included BMI^2 but the regression coefficient rounded to 0.00 so not shown in the equation.

Figure S4 Receiver Operating Characteristic curve for post-COVID breathlessness logistic regression model

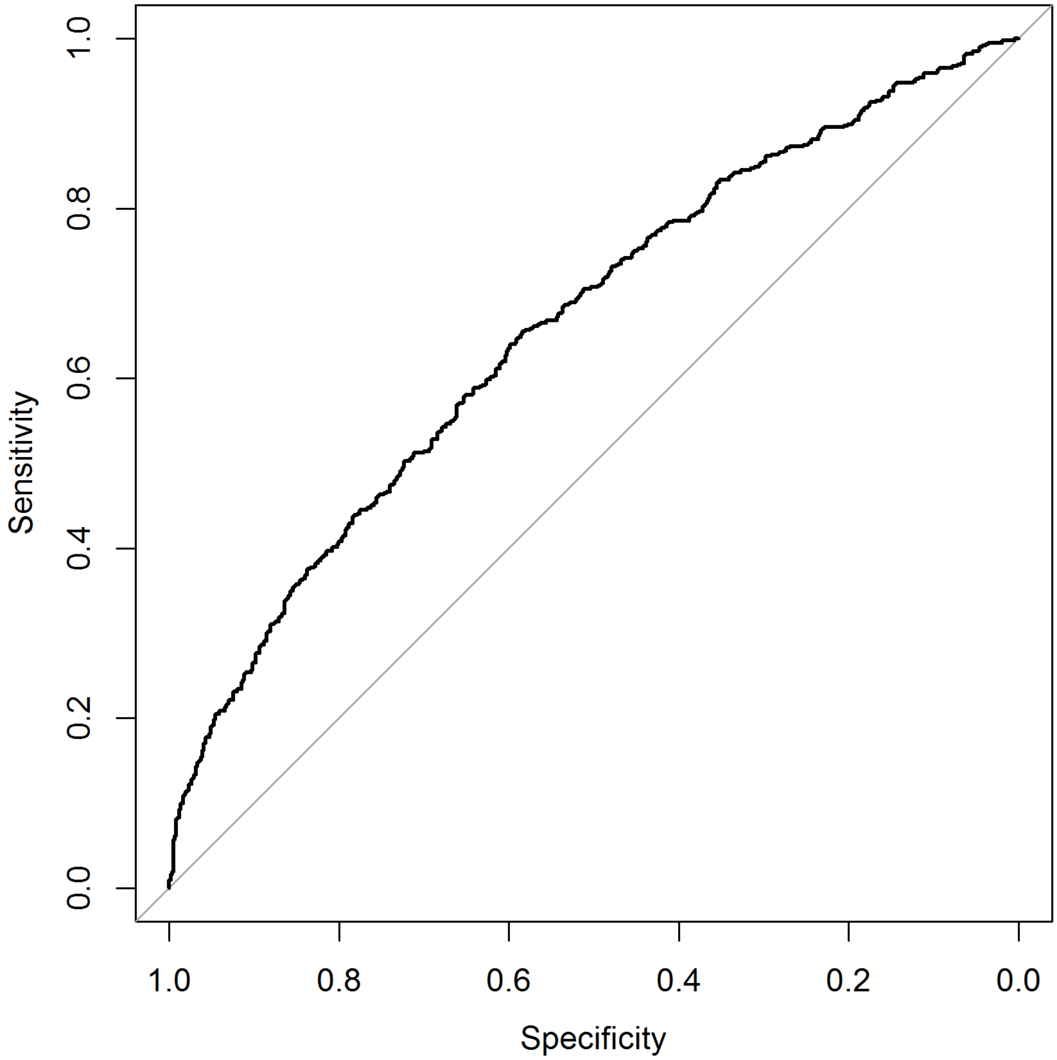

Figure S5 Calibration plot for post-COVID breathlessness logistic regression model based on the first imputed dataset

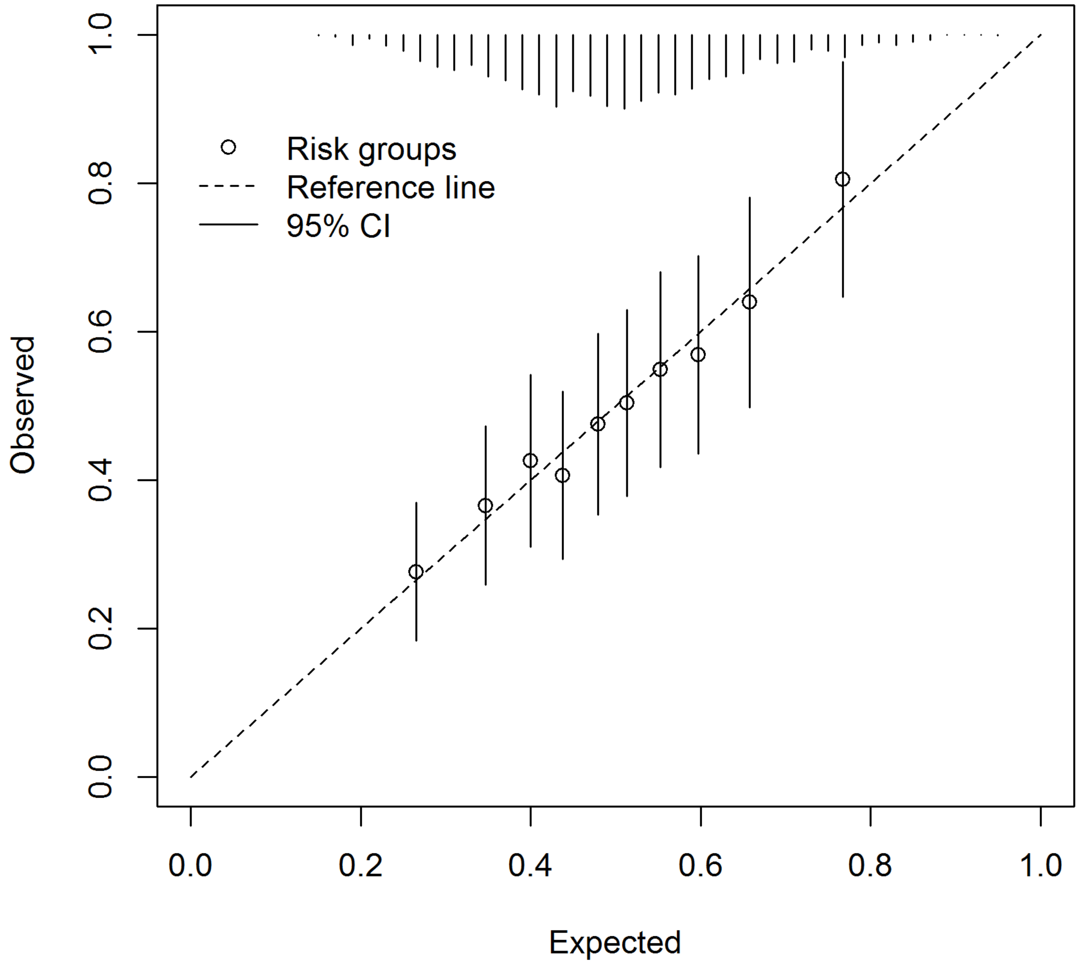

Subgroup analyses: discharge to review period

Subgroup analyses were completed based on the period between discharge and review. We grouped participants into three categories. Those who attended the research visit: within three months of discharge; between three and six months of discharge; between six to eight months after discharge. The characteristics of participants within each of the subgroups is displayed in Table S4 - Table S6. The date of discharge for each of the subgroups is displayed in Figure S6

Table S4: Patient characteristics (stratified by discharge to review period)

		Review period < 3 months			Review period 3-6 months			Review period 6-8 months		
		Post-COVID breathlessness		Total	Post-COVID breathlessness		Total	Post-COVID breathlessness		Total
		No (%)	Yes (%)		No (%)	Yes (%)		No (%)	Yes (%)	
Total N (%)		103 (47.0)	116 (53.0)	219	347 (50.0)	347 (50.0)	694	161 (51.4)	152 (48.6)	313
Age at admission (years)	<30	≤5 (-)	≤5 (-)	7 (3.2)	≤5 (-)	6 (1.7)	9 (1.3)	≤5 (-)	≤5 (-)	9 (2.9)
	30-39	≤5 (-)	9 (7.8)	12 (5.5)	15 (4.3)	23 (6.6)	38 (5.5)	20 (12.4)	9 (5.9)	29 (9.3)
	40-49	20 (19.4)	15 (12.9)	35 (16.0)	47 (13.5)	64 (18.4)	111 (16.0)	20 (12.4)	24 (15.8)	44 (14.1)
	50-59	31 (30.1)	38 (32.8)	69 (31.5)	92 (26.5)	119 (34.3)	211 (30.4)	28 (17.4)	46 (30.3)	74 (23.6)
	60-69	28 (27.2)	34 (29.3)	62 (28.3)	101 (29.1)	95 (27.4)	196 (28.2)	54 (33.5)	45 (29.6)	99 (31.6)
	70-79	15 (14.6)	14 (12.1)	29 (13.2)	67 (19.3)	31 (8.9)	98 (14.1)	25 (15.5)	16 (10.5)	41 (13.1)
	80+	≤5 (-)	≤5 (-)	5 (2.3)	19 (5.5)	6 (1.7)	25 (3.6)	7 (4.3)	≤5 (-)	10 (3.2)
	(Missing)	≤5 (-)	≤5 (-)	≤5 (-)	≤5 (-)	≤5 (-)	6 (0.9)	≤5 (-)	≤5 (-)	7 (2.2)
Sex at birth	Male	76 (73.8)	67 (57.8)	143 (65.3)	231 (66.6)	191 (55.0)	422 (60.8)	109 (67.7)	94 (61.8)	203 (64.9)
	Female	27 (26.2)	49 (42.2)	76 (34.7)	116 (33.4)	156 (45.0)	272 (39.2)	52 (32.3)	58 (38.2)	110 (35.1)
Ethnicity	White	67 (65.0)	88 (75.9)	155 (70.8)	250 (72.0)	260 (74.9)	510 (73.5)	105 (65.2)	103 (67.8)	208 (66.5)
	South Asian	15 (14.6)	11 (9.5)	26 (11.9)	43 (12.4)	41 (11.8)	84 (12.1)	24 (14.9)	19 (12.5)	43 (13.7)
	Black	10 (9.7)	7 (6.0)	17 (7.8)	23 (6.6)	16 (4.6)	39 (5.6)	19 (11.8)	17 (11.2)	36 (11.5)

	Mixed	≤5 (-)	≤5 (-)	6 (2.7)	10 (2.9)	7 (2.0)	17 (2.4)	≤5 (-)	8 (5.3)	10 (3.2)
	Other	≤5 (-)	≤5 (-)	9 (4.1)	14 (4.0)	16 (4.6)	30 (4.3)	8 (5.0)	≤5 (-)	13 (4.2)
	(Missing)	≤5 (-)	≤5 (-)	6 (2.7)	7 (2.0)	7 (2.0)	14 (2.0)	≤5 (-)	≤5 (-)	≤5 (-)
Index of multiple deprivation	1 - most deprived	20 (19.4)	29 (25.0)	49 (22.4)	65 (18.7)	83 (23.9)	148 (21.3)	27 (16.8)	42 (27.6)	69 (22.0)
	2	24 (23.3)	27 (23.3)	51 (23.3)	81 (23.3)	70 (20.2)	151 (21.8)	30 (18.6)	34 (22.4)	64 (20.4)
	3	22 (21.4)	19 (16.4)	41 (18.7)	59 (17.0)	73 (21.0)	132 (19.0)	35 (21.7)	20 (13.2)	55 (17.6)
	4	18 (17.5)	19 (16.4)	37 (16.9)	63 (18.2)	57 (16.4)	120 (17.3)	30 (18.6)	27 (17.8)	57 (18.2)
	5 - least deprived	18 (17.5)	21 (18.1)	39 (17.8)	75 (21.6)	57 (16.4)	132 (19.0)	34 (21.1)	25 (16.4)	59 (18.8)
	(Missing)	≤5 (-)	≤5 (-)	≤5 (-)	≤5 (-)	7 (2.0)	11 (1.6)	≤5 (-)	≤5 (-)	9 (2.9)
BMI	Mean (SD)	31.9 (6.5)	33.1 (7.4)	32.6 (7.0)	31.5 (7.3)	33.1 (7.5)	32.3 (7.4)	30.8 (7.1)	31.5 (5.7)	31.1 (6.5)
Smoking	Never	60 (58.3)	68 (58.6)	128 (58.4)	188 (54.2)	194 (55.9)	382 (55.0)	102 (63.4)	77 (50.7)	179 (57.2)
	Ex-smoker	41 (39.8)	47 (40.5)	88 (40.2)	147 (42.4)	141 (40.6)	288 (41.5)	58 (36.0)	72 (47.4)	130 (41.5)
	Current smoker	≤5 (-)	≤5 (-)	≤5 (-)	≤5 (-)	8 (2.3)	12 (1.7)	≤5 (-)	≤5 (-)	≤5 (-)
	(Missing)	≤5 (-)	≤5 (-)	≤5 (-)	8 (2.3)	≤5 (-)	12 (1.7)	≤5 (-)	≤5 (-)	≤5 (-)
Number of comorbidities	Median (IQR)	1.0 (0.0 to 3.0)	2.0 (1.0 to 4.0)	2.0 (0.5 to 3.0)	2.0 (1.0 to 3.0)	2.0 (1.0 to 3.5)	2.0 (1.0 to 3.0)	1.0 (0.0 to 3.0)	2.0 (0.0 to 4.0)	2.0 (0.0 to 3.0)
Cardiovascular	No	56 (54.4)	65 (56.0)	121 (55.3)	179 (51.6)	195 (56.2)	374 (53.9)	94 (58.4)	90 (59.2)	184 (58.8)
	Yes	47 (45.6)	51 (44.0)	98 (44.7)	168 (48.4)	152 (43.8)	320 (46.1)	67 (41.6)	62 (40.8)	129 (41.2)
Respiratory	No	75 (72.8)	75 (64.7)	150 (68.5)	256 (73.8)	265 (76.4)	521 (75.1)	118 (73.3)	104 (68.4)	222 (70.9)
	Yes	28 (27.2)	41 (35.3)	69 (31.5)	91 (26.2)	82 (23.6)	173 (24.9)	43 (26.7)	48 (31.6)	91 (29.1)
Depression or anxiety	No	92 (89.3)	92 (79.3)	184 (84.0)	302 (87.0)	261 (75.2)	563 (81.1)	144 (89.4)	118 (77.6)	262 (83.7)
	Yes	10 (9.7)	22 (19.0)	32 (14.6)	42 (12.1)	79 (22.8)	121 (17.4)	14 (8.7)	31 (20.4)	45 (14.4)

	(Missing)	≤5 (-)	≤5 (-)	≤5 (-)	≤5 (-)	7 (2.0)	10 (1.4)	≤5 (-)	≤5 (-)	6 (1.9)
Breathlessness before COVID-19 (PSQ)	0	57 (55.3)	78 (67.2)	135 (61.6)	216 (62.2)	232 (66.9)	448 (64.6)	101 (62.7)	97 (63.8)	198 (63.3)
	1-2	19 (18.4)	26 (22.4)	45 (20.5)	57 (16.4)	75 (21.6)	132 (19.0)	27 (16.8)	27 (17.8)	54 (17.3)
	3 or more	27 (26.2)	12 (10.3)	39 (17.8)	74 (21.3)	40 (11.5)	114 (16.4)	33 (20.5)	28 (18.4)	61 (19.5)

Table S5: Patient characteristics available during hospital admission (stratified by discharge to review period)

		Review period < 3 months			Review period 3-6 months			Review period 6-8 months		
	l	Post-COVID breathlessness			Post-COVID breathlessness			Post-COVID breathlessness		
		No (%)	Yes (%)	Total	No (%)	Yes (%)	Total	No (%)	Yes (%)	Total
Total N (%)		103 (47.0)	116 (53.0)	219	347 (50.0)	347 (50.0)	694	161 (51.4)	152 (48.6)	313
Admission duration (days)	Median (IQR)	7.0 (4.0 to 15.5)	8.5 (4.0 to 16.0)	8.0 (4.0 to 16.0)	7.0 (4.0 to 13.0)	8.0 (4.0 to 19.5)	7.0 (4.0 to 16.0)	8.0 (5.0 to 20.0)	10.5 (4.0 to 27.0)	9.0 (4.0 to 24.0)
WHO clinical progression scale	WHO – class 3-4	16 (15.5)	13 (11.2)	29 (13.2)	60 (17.3)	68 (19.6)	128 (18.4)	34 (21.1)	32 (21.1)	66 (21.1)
	WHO – class 5	49 (47.6)	54 (46.6)	103 (47.0)	160 (46.1)	129 (37.2)	289 (41.6)	43 (26.7)	42 (27.6)	85 (27.2)
	WHO – class 6	25 (24.3)	28 (24.1)	53 (24.2)	73 (21.0)	70 (20.2)	143 (20.6)	38 (23.6)	22 (14.5)	60 (19.2)
	WHO – class 7-9	13 (12.6)	21 (18.1)	34 (15.5)	54 (15.6)	80 (23.1)	134 (19.3)	46 (28.6)	56 (36.8)	102 (32.6)
Proning	No	74 (71.8)	80 (69.0)	154 (70.3)	272 (78.4)	244 (70.3)	516 (74.4)	120 (74.5)	102 (67.1)	222 (70.9)
	Yes	16 (15.5)	24 (20.7)	40 (18.3)	45 (13.0)	65 (18.7)	110 (15.9)	26 (16.1)	34 (22.4)	60 (19.2)
	(Missing)	13 (12.6)	12 (10.3)	25 (11.4)	30 (8.6)	38 (11.0)	68 (9.8)	15 (9.3)	16 (10.5)	31 (9.9)
Pulmonary Embolism	No	81 (78.6)	101 (87.1)	182 (83.1)	304 (87.6)	284 (81.8)	588 (84.7)	133 (82.6)	122 (80.3)	255 (81.5)
	Yes	14 (13.6)	8 (6.9)	22 (10.0)	26 (7.5)	41 (11.8)	67 (9.7)	16 (9.9)	16 (10.5)	32 (10.2)
	(Missing)	8 (7.8)	7 (6.0)	15 (6.8)	17 (4.9)	22 (6.3)	39 (5.6)	12 (7.5)	14 (9.2)	26 (8.3)
Coronary thrombosis	No	93 (90.3)	110 (94.8)	203 (92.7)	330 (95.1)	320 (92.2)	650 (93.7)	147 (91.3)	135 (88.8)	282 (90.1)
	Yes	≤5 (-)	≤5 (-)	≤5 (-)	≤5 (-)	≤5 (-)	≤5 (-)	≤5 (-)	≤5 (-)	≤5 (-)

	(Missing)	10 (-)	6 (-)	16 (-)	16 (-)	26 (-)	42 (-)	12 (-)	16 (-)	28 (-)
Antibiotic therapy	No	22 (21.4)	26 (22.4)	48 (21.9)	66 (19.0)	71 (20.5)	137 (19.7)	27 (16.8)	24 (15.8)	51 (16.3)
	Yes	77 (74.8)	88 (75.9)	165 (75.3)	273 (78.7)	263 (75.8)	536 (77.2)	127 (78.9)	123 (80.9)	250 (79.9)
	(Missing)	≤5 (-)	≤5 (-)	6 (2.7)	8 (2.3)	13 (3.7)	21 (3.0)	7 (4.3)	≤5 (-)	12 (3.8)
Systemic steroids (Oral or IV)	No	36 (35.0)	27 (23.3)	63 (28.8)	170 (49.0)	165 (47.6)	335 (48.3)	113 (70.2)	102 (67.1)	215 (68.7)
	Yes	60 (58.3)	86 (74.1)	146 (66.7)	158 (45.5)	160 (46.1)	318 (45.8)	32 (19.9)	35 (23.0)	67 (21.4)
	(Missing)	7 (6.8)	≤5 (-)	10 (4.6)	19 (5.5)	22 (6.3)	41 (5.9)	16 (9.9)	15 (9.9)	31 (9.9)
Therapeutic dose anti-coagulation	No	46 (44.7)	67 (57.8)	113 (51.6)	207 (59.7)	180 (51.9)	387 (55.8)	99 (61.5)	86 (56.6)	185 (59.1)
	Yes	50 (48.5)	43 (37.1)	93 (42.5)	123 (35.4)	148 (42.7)	271 (39.0)	47 (29.2)	54 (35.5)	101 (32.3)
	(Missing)	7 (6.8)	6 (5.2)	13 (5.9)	17 (4.9)	19 (5.5)	36 (5.2)	15 (9.3)	12 (7.9)	27 (8.6)
SARS-CoV-2 swab	Negative	13 (12.6)	8 (6.9)	21 (9.6)	22 (6.3)	31 (8.9)	53 (7.6)	12 (7.5)	12 (7.9)	24 (7.7)
	Positive	81 (78.6)	89 (76.7)	170 (77.6)	302 (87.0)	298 (85.9)	600 (86.5)	139 (86.3)	130 (85.5)	269 (85.9)
	(Missing)	9 (8.8)	19 (16.4)	28 (12.8)	23 (6.7)	18 (5.2)	41 (5.9)	10 (6.2)	10 (6.6)	20 (6.4)

Table S6: Patient characteristics available at the research visit (stratified by discharge to review period)

		Review period < 3 months			Review period 3-6 months			Review period 6-8 months		
		Post-COVID breathlessness			Post-COVID breathlessness			Post-COVID breathlessness		
		No (%)	Yes (%)	Total	No (%)	Yes (%)	Total	No (%)	Yes (%)	Total
Total N (%)		103 (47.0)	116 (53.0)	219	347 (50.0)	347 (50.0)	694	161 (51.4)	152 (48.6)	313
Discharge to review period (months)	Median (IQR)	2.4 (2.0 to 2.8)	2.4 (2.1 to 2.8)	2.4 (2.0 to 2.8)	4.4 (3.7 to 5.2)	4.5 (3.8 to 5.3)	4.5 (3.7 to 5.3)	6.6 (6.3 to 7.0)	6.7 (6.3 to 7.3)	6.7 (6.3 to 7.2)
Breathlessness at research visit (PSQ)	0	73 (70.9)	≤5 (-)	73 (33.3)	265 (76.4)	≤5 (-)	265 (38.2)	124 (77.0)	≤5 (-)	124 (39.6)
	1-2	12 (11.7)	28 (24.1)	40 (18.3)	39 (11.2)	86 (24.8)	125 (18.0)	19 (11.8)	29 (19.1)	48 (15.3)
	3 or more	18 (17.5)	88 (75.9)	106 (48.4)	43 (12.4)	261 (75.2)	304 (43.8)	18 (11.2)	123 (80.9)	141 (45.0)
PHQ9 total score	Median (IQR)	3.0 (0.0 to 9.0)	7.0 (3.0 to 13.0)	5.0 (2.0 to 11.0)	3.0 (1.0 to 8.0)	8.0 (3.0 to 13.0)	5.0 (2.0 to 11.0)	3.0 (1.0 to 7.0)	8.0 (3.0 to 15.0)	5.0 (1.0 to 10.0)
GAD7 total score	Median (IQR)	3.0 (0.0 to 7.0)	5.0 (0.0 to 10.0)	4.0 (0.0 to 9.0)	2.0 (0.0 to 7.0)	5.0 (1.0 to 10.0)	3.0 (0.0 to 8.0)	2.0 (0.0 to 5.0)	6.0 (2.0 to 12.2)	3.0 (0.0 to 9.0)
PCL-5 Total Severity Score	Median (IQR)	6.0 (1.0 to 17.0)	14.0 (5.0 to 32.0)	9.0 (2.5 to 26.0)	6.0 (2.0 to 15.0)	14.0 (5.0 to 30.0)	9.0 (3.0 to 23.0)	7.0 (2.0 to 15.0)	16.0 (7.0 to 39.8)	10.0 (4.0 to 23.0)
CRP	Median (IQR)	4.0 (2.0 to 5.0)	5.0 (3.4 to 6.0)	4.0 (3.0 to 5.2)	4.0 (1.5 to 5.0)	4.0 (2.0 to 5.4)	4.0 (2.0 to 5.0)	3.0 (1.0 to 5.0)	3.0 (1.0 to 5.0)	3.0 (1.0 to 5.0)
BNP/NT-Pro-BNP ng/L >threshold	No	46 (44.7)	60 (51.7)	106 (48.4)	168 (48.4)	167 (48.1)	335 (48.3)	79 (49.1)	77 (50.7)	156 (49.8)
	Yes	≤5 (-)	≤5 (-)	≤5 (-)	17 (4.9)	8 (2.3)	25 (3.6)	10 (6.2)	≤5 (-)	≤5 (-)
	(Missing)	55 (53.4)	53 (45.7)	108 (49.3)	162 (46.7)	172 (49.6)	334 (48.1)	72 (44.7)	70 (46.1)	142 (45.4)
Haemoglobin level All (g/dl)	Median (IQR)	14.6 (13.6 to 15.4)	14.1 (13.1 to 14.8)	14.4 (13.2 to 15.2)	14.3 (13.2 to 15.2)	13.9 (13.0 to 15.0)	14.1 (13.2 to 15.2)	14.3 (13.3 to 15.0)	14.1 (13.2 to 15.0)	14.2 (13.2 to 15.0)

Haemoglobin level male (g/dL)	Median (IQR)	14.8 (14.1 to 15.6)	14.6 (13.6 to 15.4)	14.8 (13.8 to 15.5)	14.7 (13.9 to 15.6)	14.6 (13.7 to 15.6)	14.7 (13.8 to 15.6)	14.7 (13.8 to 15.4)	14.6 (13.7 to 15.5)	14.6 (13.8 to 15.5)
Haemoglobin level female (g/dL)	Median (IQR)	13.6 (13.1 to 14.4)	13.2 (12.8 to 14.1)	13.4 (13.0 to 14.2)	13.5 (12.8 to 14.4)	13.4 (12.6 to 13.9)	13.4 (12.6 to 14.1)	13.3 (12.1 to 13.9)	13.4 (12.2 to 14.0)	13.3 (12.1 to 14.0)
ISWT distance (m)	Median (IQR)	390.0 (270.0 to 550.0)	345.0 (222.5 to 540.0)	360.0 (245.0 to 550.0)	440.0 (267.5 to 622.5)	350.0 (222.5 to 550.0)	380.0 (250.0 to 569.5)	454.0 (322.5 to 622.5)	350.0 (260.0 to 532.2)	415.0 (270.0 to 600.0)
ISWT % predicted	Median (IQR)	54.8 (30.2 to 71.4)	53.5 (32.3 to 69.2)	54.0 (32.3 to 69.3)	61.7 (43.1 to 81.9)	50.4 (34.6 to 69.5)	56.4 (38.2 to 75.8)	62.8 (46.4 to 89.8)	53.8 (37.8 to 73.4)	58.7 (39.2 to 77.7)
Oxygen saturations post ISWT (%)	Median (IQR)	96.0 (94.0 to 98.0)	96.0 (94.0 to 97.0)	96.0 (94.0 to 97.0)	96.0 (94.0 to 97.0)	96.0 (93.0 to 97.0)	96.0 (93.0 to 97.0)	97.0 (94.0 to 99.0)	96.0 (94.0 to 98.0)	97.0 (94.0 to 98.2)
Borg leg fatigue score post ISWT	Median (IQR)	2.0 (0.5 to 3.0)	3.0 (0.5 to 3.0)	2.0 (0.5 to 3.0)	2.0 (0.5 to 4.0)	3.0 (2.0 to 4.0)	3.0 (1.0 to 4.0)	2.0 (0.5 to 3.0)	3.0 (2.0 to 5.0)	3.0 (1.0 to 4.0)
FEV1 (L)	Median (IQR)	2.7 (2.4 to 3.2)	2.6 (2.1 to 3.1)	2.6 (2.2 to 3.2)	2.8 (2.3 to 3.3)	2.7 (2.2 to 3.3)	2.8 (2.2 to 3.3)	2.9 (2.2 to 3.4)	2.6 (2.1 to 3.3)	2.7 (2.1 to 3.4)
FEV1 % predicted	Median (IQR)	94.2 (86.4 to 107.1)	87.2 (79.5 to 97.1)	89.9 (81.2 to 101.6)	93.9 (82.9 to 105.2)	90.9 (77.9 to 101.6)	92.1 (79.6 to 103.4)	93.5 (82.8 to 105.5)	90.0 (77.7 to 101.8)	92.0 (79.2 to 104.3)
FEV1 < LLN	No	32 (84.2)	50 (80.6)	82 (82.0)	158 (87.8)	155 (77.9)	313 (82.6)	84 (81.6)	79 (78.2)	163 (79.9)
	Yes	6 (15.8)	12 (19.4)	18 (18.0)	22 (12.2)	44 (22.1)	66 (17.4)	19 (18.4)	22 (21.8)	41 (20.1)
FVC	Median (IQR)	3.5 (2.9 to 4.3)	3.1 (2.5 to 3.9)	3.4 (2.7 to 4.1)	3.6 (3.0 to 4.3)	3.4 (2.7 to 4.1)	3.5 (2.8 to 4.2)	3.5 (2.8 to 4.4)	3.2 (2.5 to 4.1)	3.4 (2.6 to 4.3)
FVC % predicted	Median (IQR)	92.1 (83.7 to 104.9)	84.1 (75.8 to 96.4)	87.4 (79.5 to 99.7)	95.3 (83.5 to 105.1)	88.2 (75.7 to 99.6)	91.3 (79.4 to 102.6)	91.9 (78.3 to 106.2)	84.4 (72.5 to 98.5)	88.8 (75.1 to 102.7)
FVC < LLN	No	34 (89.5)	45 (72.6)	79 (79.0)	158 (87.8)	145 (72.9)	303 (79.9)	84 (82.4)	70 (70.0)	154 (76.2)
	Yes	≤5 (-)	17 (27.4)	21 (21.0)	22 (12.2)	54 (27.1)	76 (20.1)	18 (17.6)	30 (30.0)	48 (23.8)

FEV1/FVC ratio expressed as %	Median (IQR)	79.7 (75.1 to 85.5)	82.4 (78.0 to 85.4)	81.0 (76.7 to 85.6)	77.8 (72.6 to 82.6)	80.8 (77.1 to 85.5)	79.5 (75.3 to 84.4)	81.8 (76.2 to 85.6)	83.6 (78.7 to 89.1)	82.5 (77.3 to 87.8)
FEV1/FVC < LLN	No	34 (91.9)	61 (98.4)	95 (96.0)	165 (92.2)	190 (96.4)	355 (94.4)	96 (95.0)	91 (93.8)	187 (94.4)
	Yes	≤5 (-)	≤5 (-)	≤5 (-)	14 (7.8)	7 (3.6)	21 (5.6)	≤5 (-)	6 (6.2)	11 (5.6)
TLCO	Median (IQR)	7.7 (6.7 to 8.4)	6.4 (5.2 to 8.1)	7.0 (6.0 to 8.2)	7.8 (6.8 to 9.3)	6.9 (5.7 to 8.2)	7.4 (6.1 to 8.7)	7.0 (5.7 to 7.7)	6.7 (6.2 to 8.6)	6.9 (5.9 to 8.3)
TLCO % predicted	Median (IQR)	93.8 (83.0 to 101.5)	90.6 (68.1 to 101.7)	90.7 (74.6 to 101.9)	94.6 (80.8 to 103.9)	87.5 (73.1 to 104.1)	91.4 (75.4 to 104.0)	86.8 (77.5 to 94.9)	93.6 (86.2 to 106.1)	89.7 (78.4 to 99.1)
TLCO predicted <80%	No	16 (76.2)	20 (64.5)	36 (69.2)	48 (76.2)	45 (63.4)	93 (69.4)	22 (62.9)	25 (80.6)	47 (71.2)
	Yes	≤5 (-)	11 (35.5)	16 (30.8)	15 (23.8)	26 (36.6)	41 (30.6)	13 (37.1)	6 (19.4)	19 (28.8)
KCO	Median (IQR)	1.5 (1.5 to 1.7)	1.5 (1.2 to 1.6)	1.5 (1.2 to 1.7)	1.5 (1.3 to 1.6)	1.5 (1.3 to 1.6)	1.5 (1.3 to 1.6)	1.4 (1.2 to 1.5)	1.5 (1.3 to 1.7)	1.4 (1.2 to 1.6)
KCO % predicted	Median (IQR)	105.4 (97.2 to 108.8)	97.9 (85.6 to 111.3)	102.5 (92.9 to 111.1)	104.9 (92.7 to 112.2)	99.6 (86.6 to 111.3)	102.3 (89.2 to 112.2)	99.8 (90.7 to 105.5)	100.3 (87.5 to 115.8)	100.3 (87.5 to 108.1)
KCO predicted <80%	No	20 (95.2)	28 (84.8)	48 (88.9)	59 (92.2)	67 (93.1)	126 (92.6)	33 (91.7)	32 (97.0)	65 (94.2)
	Yes	≤5 (-)	≤5 (-)	6 (11.1)	≤5 (-)	≤5 (-)	10 (7.4)	≤5 (-)	≤5 (-)	≤5 (-)

Figure S6: Discharge date of study participants stratified by the period between discharge and research visit

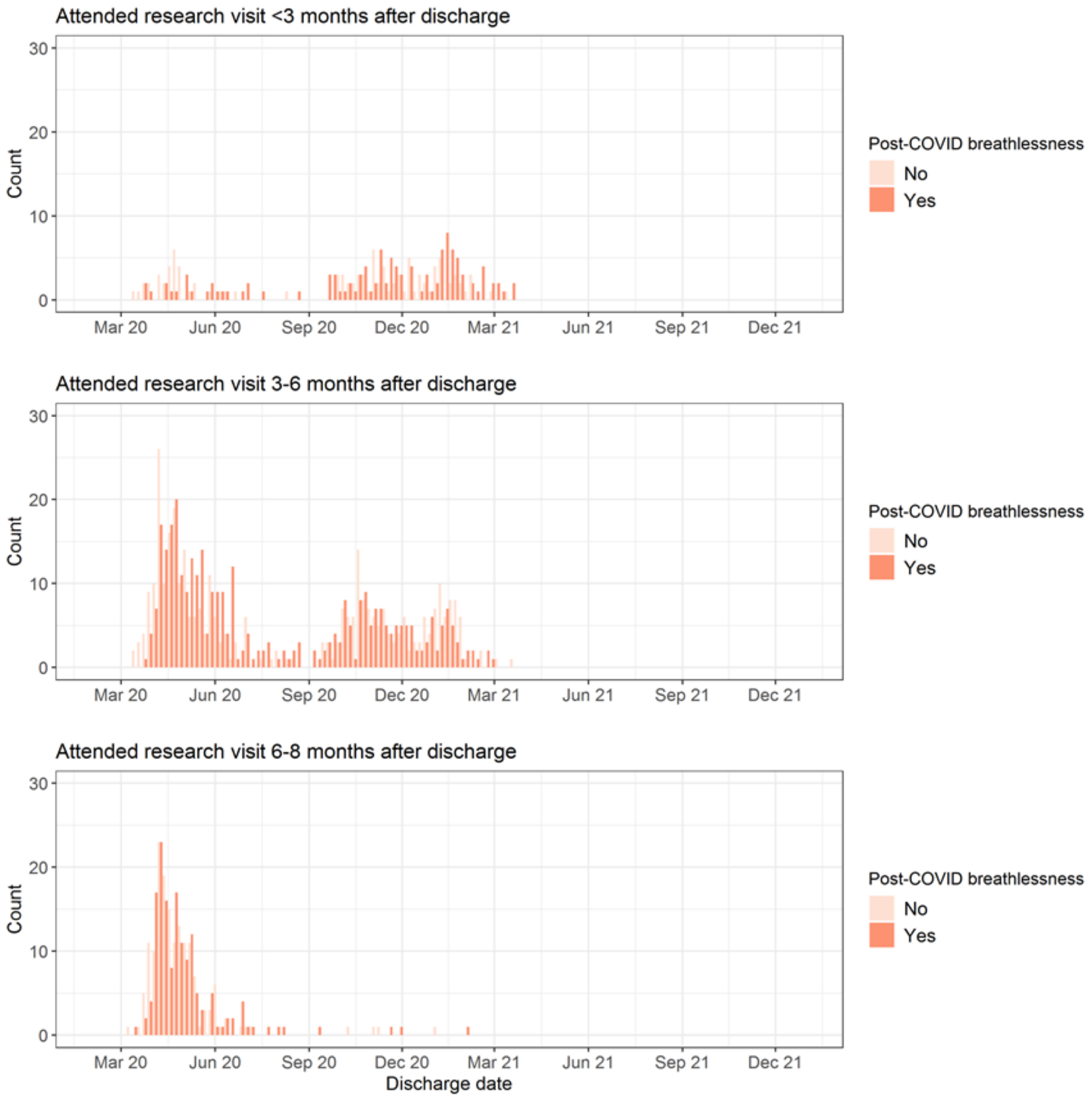

Sensitivity analyses: PSQ breathlessness at the research visit as a linear outcome

We ran a linear regression model using a complete case analysis for PSQ breathlessness score at the research visit modelled as a linear outcome (0-10). Consistent with results from the primary outcome, the most deprived quintiles, female sex and admission duration were associated with PSQ breathlessness at the research visit, though not pre-existing anxiety/depression. Participants aged 60 years or older, but not individuals of Black ethnicity were less likely to report breathlessness than the reference group. The number of co-morbidities and PSQ breathlessness before admission were also associated with breathlessness at the research visit when modelled as a linear outcome.

Table S7 Linear regression model using breathlessness at research visit (PSQ) as the dependent variable

Dependent: Breathlessness		value	Coefficient (univariable)	Coefficient (multivariable)
Sex at birth	Male	2.4 (2.8)	-	-
	Female	3.2 (3.0)	0.81 (0.47 to 1.14, p<0.001)	0.52 (0.17 to 0.86, p=0.003)
Age at admission (years)	50-59	3.1 (3.0)	-	-
	<30	2.6 (2.9)	-0.52 (-1.69 to 0.65, p=0.384)	-0.37 (-1.52 to 0.79, p=0.534)
	30-39	2.2 (2.6)	-0.78 (-1.49 to -0.08, p=0.030)	-0.59 (-1.29 to 0.10, p=0.095)
	40-49	2.6 (2.8)	-0.61 (-1.12 to -0.10, p=0.019)	-0.35 (-0.86 to 0.16, p=0.178)
	60-69	2.8 (2.9)	-0.31 (-0.73 to 0.12, p=0.157)	-0.73 (-1.16 to -0.29, p=0.001)
	70-79	2.3 (2.7)	-0.83 (-1.36 to -0.30, p=0.002)	-1.23 (-1.79 to -0.68, p<0.001)
	80+	2.3 (3.0)	-0.61 (-1.56 to 0.33, p=0.205)	-1.10 (-2.07 to -0.13, p=0.027)
Index of Multiple Deprivation	5 - least deprived	2.2 (2.5)	-	-
	4	2.3 (2.7)	0.15 (-0.39 to 0.68, p=0.588)	0.40 (-0.13 to 0.93, p=0.142)
	3	2.8 (2.9)	0.52 (-0.01 to 1.04, p=0.055)	0.52 (-0.00 to 1.04, p=0.051)
	2	2.8 (2.9)	0.68 (0.18 to 1.19, p=0.008)	0.66 (0.15 to 1.17, p=0.011)
	1 - most deprived	3.3 (3.1)	1.20 (0.69 to 1.71, p<0.001)	1.15 (0.63 to 1.67, p<0.001)
Ethnicity	White	2.8 (2.9)	-	-
	South Asian	2.5 (2.9)	-0.40 (-0.90 to 0.09, p=0.111)	-0.01 (-0.55 to 0.53, p=0.962)
	Black	2.5 (3.2)	-0.34 (-0.96 to 0.28, p=0.287)	-0.44 (-1.07 to 0.19, p=0.172)
	Mixed	2.4 (2.9)	-0.15 (-1.16 to 0.85, p=0.763)	-0.13 (-1.14 to 0.88, p=0.804)
	Other	2.5 (2.7)	-0.44 (-1.25 to 0.37, p=0.288)	-0.29 (-1.12 to 0.54, p=0.498)
BMI	[16.5,77.8]	2.7 (2.9)	0.06 (0.04 to 0.08, p<0.001)	0.11 (-0.02 to 0.24, p=0.091)
Number of comorbidities	[0.0,17.0]	2.7 (2.9)	0.33 (0.25 to 0.40, p<0.001)	0.13 (0.02 to 0.23, p=0.017)
Pre-existing depression or anxiety	No	2.5 (2.8)	-	-
	Yes	3.9 (2.9)	1.51 (1.07 to 1.94, p<0.001)	0.52 (-0.01 to 1.05, p=0.055)
Pre-existing respiratory condition	No	2.4 (2.8)	-	-
	Yes	3.6 (3.0)	1.13 (0.77 to 1.49, p<0.001)	0.22 (-0.19 to 0.63, p=0.293)
Smoking	Never	2.5 (2.8)	-	-
	Ex-smoker	3.0 (3.0)	0.54 (0.21 to 0.87, p=0.001)	0.28 (-0.06 to 0.62, p=0.111)
	Current smoker	3.5 (3.0)	0.96 (-0.39 to 2.32, p=0.162)	0.09 (-1.32 to 1.50, p=0.902)
Breathlessness before admission (PSQ)	[0.0,10.0]	2.7 (2.9)	0.47 (0.39 to 0.54, p<0.001)	0.40 (0.31 to 0.50, p<0.001)
Admission duration (days)	[0.0,171.0]	2.7 (2.9)	0.02 (0.01 to 0.02, p<0.001)	0.01 (0.00 to 0.03, p=0.006)
WHO clinical progression scale	WHO – class 3-4	2.8 (2.9)	-	-
	WHO – class 5	2.5 (2.8)	-0.17 (-0.63 to 0.29, p=0.473)	-0.17 (-0.65 to 0.30, p=0.477)
	WHO – class 6	2.6 (2.8)	-0.19 (-0.71 to 0.33, p=0.479)	-0.33 (-0.88 to 0.21, p=0.229)
	WHO – class 7-9	3.1 (3.0)	0.29 (-0.23 to 0.80, p=0.277)	0.20 (-0.45 to 0.85, p=0.549)

BMI = Body Mass Index. IV = Intravenous. World Health Organization (WHO) clinical progression scale: not requiring continuous supplemental oxygen (levels 3-4); continuous supplemental oxygen only (level 5); Continuous Positive Airway Pressure (CPAP) ventilation, Bi-Level Positive Airway Pressure or High Flow Nasal Oxygen (HFNO) (level 6); Invasive Mechanical Ventilation (IMV), Extra-Corporeal Membrane Oxygenation (ECMO) and acute Renal Replacement Therapy (RRT) (levels 7-9).

Sensitivity analyses: PSQ breathlessness at the research visit as a binary outcome

As a second sensitivity analysis, the PSQ breathlessness score reported at the research visit was categorised into a binary variable (taking a PSQ of less than 3 as "not breathless" and a PSQ of greater or equal to 3 as a "breathless") and used as the outcome variable for a multivariable logistic regression using a complete case analysis. For consistency, we used the same explanatory variables as selected for the primary outcome, though we accounted for other characteristics likely to influence breathlessness, smoking status, and breathlessness prior to COVID-19 recorded by the PSQ. Patient characteristics stratified by PSQ breathlessness at the research visit are available in Table S8-Table S10. Results of the Multivariable logistic regression with breathlessness at the research visit as the dependent variable are displayed in Table S11.

Table S8 Patient characteristics stratified by breathlessness at the research visit (PSQ)

	Total N	Levels	Not breathless	Breathless	Total
Total N (%)			675 (55.1)	551 (44.9)	1226
Age at admission (years)	1213 (98.9)	<30	15 (2.2)	10 (1.8)	25 (2.0)
		30-39	47 (7.0)	32 (5.8)	79 (6.4)
		40-49	115 (17.0)	75 (13.6)	190 (15.5)
		50-59	176 (26.1)	178 (32.3)	354 (28.9)
		60-69	191 (28.3)	166 (30.1)	357 (29.1)
		70-79	102 (15.1)	66 (12.0)	168 (13.7)
		80+	22 (3.3)	18 (3.3)	40 (3.3)
		(Missing)	7 (1.0)	6 (1.1)	13 (1.1)
Sex at birth	1226 (100.0)	Male	459 (68.0)	309 (56.1)	768 (62.6)
		Female	216 (32.0)	242 (43.9)	458 (37.4)
Ethnicity	1203 (98.1)	White	456 (67.6)	417 (75.7)	873 (71.2)
		South Asian	95 (14.1)	58 (10.5)	153 (12.5)
		Black	58 (8.6)	34 (6.2)	92 (7.5)
		Mixed	19 (2.8)	14 (2.5)	33 (2.7)
		Other	34 (5.0)	18 (3.3)	52 (4.2)
		(Missing)	13 (1.9)	10 (1.8)	23 (1.9)
Index of multiple deprivation	1204 (98.2)	1 - most deprived	125 (18.5)	141 (25.6)	266 (21.7)
		2	141 (20.9)	125 (22.7)	266 (21.7)
		3	128 (19.0)	100 (18.1)	228 (18.6)
		4	131 (19.4)	83 (15.1)	214 (17.5)
		5 - least deprived	141 (20.9)	89 (16.2)	230 (18.8)
		(Missing)	9 (1.3)	13 (2.4)	22 (1.8)
BMI	1090 (88.9)	Mean (SD)	30.9 (6.6)	33.3 (7.5)	32.0 (7.1)
Smoking	1213 (98.9)	Never	407 (60.3)	282 (51.2)	689 (56.2)
		Ex-smoker	253 (37.5)	253 (45.9)	506 (41.3)
		Current smoker	8 (1.2)	10 (1.8)	18 (1.5)
Number of comorbidities	1226 (100.0)	Median (IQR)	1.0 (0.0 to 3.0)	2.0 (1.0 to 4.0)	2.0 (0.0 to 3.0)
Pre-existing cardiovascular condition	1226 (100.0)	No	391 (57.9)	288 (52.3)	679 (55.4)
		Yes	284 (42.1)	263 (47.7)	547 (44.6)
Pre-existing respiratory condition	1226 (100.0)	No	534 (79.1)	359 (65.2)	893 (72.8)
		Yes	141 (20.9)	192 (34.8)	333 (27.2)
Depression or anxiety	1207 (98.5)	No	598 (88.6)	411 (74.6)	1009 (82.3)
		Yes	67 (9.9)	131 (23.8)	198 (16.2)

Breathlessness at the research visit was taken as a PSQ <3 as "not breathless" and a PSQ of ≥ to 3 as "breathless". BMI = Body Mass Index.

Table S9 Patient characteristics available during hospital admission, stratified by breathlessness at the research visit (PSQ)

	Total N	Levels	Not breathless	Breathless	Total
Total N (%)			675 (55.1)	551 (44.9)	1226
Admission duration (days)	1225 (99.9)	Median (IQR)	7.0 (4.0 to 14.0)	9.0 (4.0 to 21.0)	8.0 (4.0 to 17.0)
WHO clinical progression scale	1226 (100.0)	WHO – class 3-4	126 (18.7)	97 (17.6)	223 (18.2)
		WHO – class 5	271 (40.1)	206 (37.4)	477 (38.9)
		WHO – class 6	144 (21.3)	112 (20.3)	256 (20.9)
		WHO – class 7-9	134 (19.9)	136 (24.7)	270 (22.0)
Proning during mechanical ventilation	1102 (89.9)	No	508 (75.3)	384 (69.7)	892 (72.8)
		Yes	104 (15.4)	106 (19.2)	210 (17.1)
		(Missing)	63 (9.3)	61 (11.1)	124 (10.1)
Pulmonary Embolism	1146 (93.5)	No	569 (84.3)	456 (82.8)	1025 (83.6)
		Yes	63 (9.3)	58 (10.5)	121 (9.9)
		(Missing)	43 (6.4)	37 (6.7)	80 (6.5)
Coronary thrombosis	1140 (93.0)	No	629 (93.2)	506 (91.8)	1135 (92.6)
		Yes	<5 (-)	<5 (-)	5 (0.4)
		(Missing)	- (-)	- (-)	86 (7.0)
Antibiotic therapy	1187 (96.8)	No	135 (20.0)	101 (18.3)	236 (19.2)
		Yes	518 (76.7)	433 (78.6)	951 (77.6)
		(Missing)	22 (3.3)	17 (3.1)	39 (3.2)
Systemic steroids (Oral or IV)	1144 (93.3)	No	353 (52.3)	260 (47.2)	613 (50.0)
		Yes	278 (41.2)	253 (45.9)	531 (43.3)
		(Missing)	44 (6.5)	38 (6.9)	82 (6.7)
Therapeutic dose anti-coagulation	1010 (93.8)	No	349 (58.1)	276 (58.0)	625 (58.0)
		Yes	216 (35.9)	169 (35.5)	385 (35.7)
		(Missing)	36 (6.0)	31 (6.5)	67 (6.2)

Breathlessness at the research visit was taken as a PSQ <3 as “not breathless” and a PSQ of ≥ to 3 as “breathless”. IV = Intravenous. World Health Organization (WHO) clinical progression scale: not requiring continuous supplemental oxygen (levels 3-4); continuous supplemental oxygen only (level 5); Continuous Positive Airway Pressure (CPAP) ventilation, Bi-Level Positive Airway Pressure or High Flow Nasal Oxygen (HFNO) (level 6); Invasive Mechanical Ventilation (IMV), Extra-Corporeal Membrane Oxygenation (ECMO) and acute Renal Replacement Therapy (RRT) (levels 7-9). Cell counts <5 and related sub-totals have been suppressed.

Table S10 Patient characteristics available at the research visit, stratified by breathlessness at the research visit (PSQ)

	Total N	Levels	Not breathless	Breathless	Total
Total N (%)			675 (55.1)	551 (44.9)	1226
Discharge to review period (months)	1226 (100.0)	Median (IQR)	4.8 (3.4 to 6.0)	4.5 (3.3 to 6.0)	4.7 (3.4 to 6.0)
PHQ9 total score	1188 (96.9)	Median (IQR)	3.0 (1.0 to 7.0)	9.0 (4.0 to 15.0)	5.0 (2.0 to 11.0)
GAD7 total score	1187 (96.8)	Median (IQR)	2.0 (0.0 to 6.0)	6.0 (1.0 to 12.0)	3.0 (0.0 to 8.0)
PCL-5 Total Severity Score	1184 (96.6)	Median (IQR)	6.0 (2.0 to 14.0)	18.0 (7.0 to 35.0)	9.0 (3.0 to 23.0)
CRP	800 (65.3)	Median (IQR)	4.0 (1.1 to 5.0)	4.0 (2.0 to 5.7)	4.0 (1.8 to 5.0)
BNP/NT-Pro-BNP ng/L >threshold	642 (52.4)	No	330 (48.9)	267 (48.5)	597 (48.7)
		Yes	21 (3.1)	24 (4.4)	45 (3.7)
		(Missing)	324 (48.0)	260 (47.2)	584 (47.6)
Haemoglobin level All (g/dL)	861 (70.2)	Median (IQR)	14.4 (13.4 to 15.3)	13.9 (13.0 to 14.9)	14.2 (13.2 to 15.2)
Haemoglobin level male (g/dL)	537 (43.8)	Median (IQR)	14.8 (13.9 to 15.6)	14.5 (13.6 to 15.5)	14.7 (13.8 to 15.5)
Haemoglobin level female (g/dL)	324 (26.4)	Median (IQR)	13.5 (12.9 to 14.2)	13.3 (12.6 to 14.0)	13.4 (12.7 to 14.1)
ISWT distance (m)	737 (60.1)	Median (IQR)	460.0 (300.0 to 650.0)	320.0 (200.0 to 460.0)	380.0 (257.5 to 570.0)
ISWT % predicted	658 (53.7)	Median (IQR)	62.4 (45.4 to 84.5)	48.1 (30.8 to 67.8)	56.3 (37.9 to 75.9)
Oxygen saturations post ISWT (%)	727 (59.3)	Median (IQR)	96.0 (94.0 to 98.0)	96.0 (93.0 to 98.0)	96.0 (94.0 to 98.0)
Borg leg fatigue score post ISWT	722 (58.9)	Median (IQR)	2.0 (0.5 to 3.0)	3.0 (2.0 to 5.0)	3.0 (1.0 to 4.0)
FEV1 (L)	748 (61.0)	Median (IQR)	2.9 (2.5 to 3.4)	2.5 (2.0 to 3.2)	2.8 (2.2 to 3.3)
FEV1 % predicted	683 (55.7)	Median (IQR)	95.1 (85.3 to 106.8)	87.2 (74.9 to 99.3)	91.7 (79.7 to 103.7)
FEV1 < LLN	683 (55.7)	No	321 (88.4)	237 (74.1)	558 (81.7)
		Yes	42 (11.6)	83 (25.9)	125 (18.3)
FVC (L)	746 (60.8)	Median (IQR)	3.6 (3.0 to 4.4)	3.1 (2.5 to 3.9)	3.5 (2.8 to 4.2)
FVC % predicted	681 (55.5)	Median (IQR)	94.0 (83.4 to 105.7)	84.8 (73.1 to 97.3)	90.0 (78.2 to 102.4)
FVC < LLN	681 (55.5)	No	316 (87.5)	220 (68.8)	536 (78.7)
		Yes	45 (12.5)	100 (31.2)	145 (21.3)
FEV1/FVC expressed as a %	736 (60.0)	Median (IQR)	80.0 (75.7 to 84.8)	81.0 (76.4 to 85.9)	80.6 (76.0 to 85.5)
FEV1/FVC < LLN	673 (54.9)	No	341 (95.5)	296 (93.7)	637 (94.7)
		Yes	16 (4.5)	20 (6.3)	36 (5.3)
TLCO	272 (22.2)	Median (IQR)	7.7 (6.6 to 8.9)	6.5 (5.5 to 8.1)	7.3 (6.1 to 8.4)
TLCO % predicted	252 (20.6)	Median (IQR)	93.5 (80.0 to 105.0)	88.1 (74.0 to 100.4)	90.7 (76.8 to 103.2)
TLCO predicted <80%	252 (20.6)	No	101 (74.8)	75 (64.1)	176 (69.8)
		Yes	34 (25.2)	42 (35.9)	76 (30.2)

	Total N	Levels	Not breathless	Breathless	Total
KCO	276 (22.5)	Median (IQR)	1.5 (1.3 to 1.6)	1.5 (1.2 to 1.7)	1.5 (1.3 to 1.6)
KCO % predicted	259 (21.1)	Median (IQR)	103.0 (91.9 to 108.9)	100.2 (86.8 to 112.6)	101.8 (89.2 to 110.1)
KCO predicted <80%	259 (21.1)	No	124 (91.9)	115 (92.7)	239 (92.3)
		Yes	11 (8.1)	9 (7.3)	20 (7.7)

Breathlessness at the research visit was taken as a PSQ <3 as "not breathless" and a PSQ of ≥ to 3 as a "breathless". PHQ-9 = Patient Health Questionnaire-9, GAD-7 = General Anxiety Disorder-7, PCL-5 = Posttraumatic Stress Disorder Checklist, CRP = C-reactive Protein, BNP = Brain Natriuretic Peptide, NT-Pro-BNP = N-terminal-pro hormone BNP, ISWT = Incremental Shuttle Walk Test, FEV1 = Forced Expiratory Volume in 1 second, LLN = Lower Limit of Normal, FVC = Forced Vital Capacity, TLCO = Transfer Capacity of the lung, KCO = Carbon monoxide transfer coefficient, PSQ = Patient Symptom Questionnaire.

Table S11 Multivariable logistic regression with breathlessness at research visit (PSQ) as the dependent variable

Consistent with results from the primary outcome, the most deprived quintile, female sex, pre-existing depression/anxiety, and admission duration were associated with PSQ breathlessness at the research visit. Individuals of Black ethnicity and participants aged 60 years or older were less likely to report breathlessness than the reference group. PSQ breathlessness before admission was also associated with PSQ breathlessness at the research visit.

Dependent: Breathlessness at research visit (PSQ)		Not breathless	Breathless	OR (univariable)	OR (multivariable)
Sex at birth	Male	459 (59.8)	309 (40.2)	-	-
	Female	216 (47.2)	242 (52.8)	1.66 (1.32-2.10, p<0.001)	1.37 (1.03-1.83, p=0.032)
Age at admission (years)	50-59	176 (49.7)	178 (50.3)	-	-
	<30	15 (60.0)	10 (40.0)	0.66 (0.28-1.49, p=0.323)	0.71 (0.25-1.94, p=0.516)
	30-39	47 (59.5)	32 (40.5)	0.67 (0.41-1.10, p=0.117)	0.72 (0.40-1.28, p=0.273)
	40-49	115 (60.5)	75 (39.5)	0.64 (0.45-0.92, p=0.016)	0.81 (0.53-1.24, p=0.335)
	60-69	191 (53.5)	166 (46.5)	0.86 (0.64-1.15, p=0.313)	0.68 (0.47-0.97, p=0.035)
	70-79	102 (60.7)	66 (39.3)	0.64 (0.44-0.93, p=0.019)	0.51 (0.31-0.81, p=0.005)
	80+	22 (55.0)	18 (45.0)	0.81 (0.42-1.56, p=0.527)	0.67 (0.29-1.53, p=0.343)
Index of Multiple Deprivation	5 - least deprived	141 (61.3)	89 (38.7)	-	-
	4	131 (61.2)	83 (38.8)	1.00 (0.68-1.47, p=0.985)	1.27 (0.81-1.99, p=0.302)
	3	128 (56.1)	100 (43.9)	1.24 (0.85-1.80, p=0.262)	1.30 (0.84-2.03, p=0.241)
	2	141 (53.0)	125 (47.0)	1.40 (0.98-2.01, p=0.063)	1.52 (0.99-2.36, p=0.056)
	1 - most deprived	125 (47.0)	141 (53.0)	1.79 (1.25-2.56, p=0.001)	1.95 (1.26-3.04, p=0.003)
Ethnicity	White	456 (52.2)	417 (47.8)	-	-
	South Asian	95 (62.1)	58 (37.9)	0.67 (0.47-0.95, p=0.025)	0.87 (0.55-1.37, p=0.560)
	Black	58 (63.0)	34 (37.0)	0.64 (0.41-0.99, p=0.049)	0.58 (0.34-0.99, p=0.049)
	Mixed	19 (57.6)	14 (42.4)	0.81 (0.39-1.62, p=0.547)	0.76 (0.31-1.79, p=0.536)
	Other	34 (65.4)	18 (34.6)	0.58 (0.32-1.03, p=0.068)	0.54 (0.25-1.10, p=0.096)
BMI	Mean (SD)	30.9 (6.6)	33.3 (7.5)	1.05 (1.03-1.07, p<0.001)	1.12 (1.00-1.26, p=0.052)
Number of comorbidities	Mean (SD)	1.7 (1.8)	2.6 (2.4)	1.24 (1.16-1.32, p<0.001)	1.05 (0.96-1.16, p=0.285)
Pre-existing depression or anxiety	No	598 (59.3)	411 (40.7)	-	-
	Yes	67 (33.8)	131 (66.2)	2.84 (2.07-3.94, p<0.001)	1.72 (1.10-2.71, p=0.019)
Pre-existing respiratory condition	No	534 (59.8)	359 (40.2)	-	-
	Yes	141 (42.3)	192 (57.7)	2.03 (1.57-2.62, p<0.001)	1.19 (0.85-1.68, p=0.307)
Smoking	Never	407 (59.1)	282 (40.9)	-	-
	Ex-smoker	253 (50.0)	253 (50.0)	1.44 (1.15-1.82, p=0.002)	1.21 (0.91-1.61, p=0.198)
	Current smoker	8 (44.4)	10 (55.6)	1.80 (0.70-4.78, p=0.220)	1.17 (0.36-3.94, p=0.795)
Breathlessness before admission (PSQ)	Mean (SD)	0.6 (1.5)	1.8 (2.4)	1.37 (1.28-1.47, p<0.001)	1.33 (1.22-1.45, p<0.001)
Admission duration (days)	Mean (SD)	13.5 (18.1)	17.3 (21.8)	1.01 (1.00-1.02, p=0.001)	1.01 (1.00-1.02, p=0.024)
WHO clinical progression scale	WHO – class 3-4	126 (56.5)	97 (43.5)	-	-
	WHO – class 5	271 (56.8)	206 (43.2)	0.99 (0.72-1.36, p=0.938)	0.86 (0.58-1.30, p=0.478)
	WHO – class 6	144 (56.2)	112 (43.8)	1.01 (0.70-1.45, p=0.956)	0.88 (0.55-1.39, p=0.570)
	WHO – class 7-9	134 (49.6)	136 (50.4)	1.32 (0.92-1.88, p=0.128)	1.18 (0.68-2.03, p=0.553)

Dependent: Breathlessness at research visit (PSQ)	Not breathless	Breathless	OR (univariable)	OR (multivariable)

BMI = Body Mass Index. PSQ = Patient Symptom Questionnaire. World Health Organization (WHO) clinical progression scale: not requiring continuous supplemental oxygen (levels 3-4); continuous supplemental oxygen only (level 5); Continuous Positive Airway Pressure (CPAP) ventilation, Bi-Level Positive Airway Pressure or High Flow Nasal Oxygen (HFNO) (level 6); Invasive Mechanical Ventilation (IMV), Extra-Corporeal Membrane Oxygenation (ECMO) and acute Renal Replacement Therapy (RRT) (levels 7-9). The logistic regression model was also adjusted for BMI^2. Breathlessness at the research visit was taken as a PSQ <3 as "not breathless" and a PSQ of ≥ to 3 as "breathless".

Sensitivity analyses: PSQ breathlessness at the research visit assessed in a multinomial model
As a further sensitivity analysis, we repeated the multinomial modelling using the PSQ breathlessness score reported at the research visit as the outcome (taking a PSQ of less than 3 as "not breathless"; a PSQ 3 – 6 as "mildly breathless" and a PSQ of greater or equal to 7 as a "severely breathless") as a complete case analysis.

The most deprived quintiles, female sex, and admission duration were associated with *severe* breathlessness at the research visit, in addition to number of co-morbidities. Pre-existing depression/anxiety and pre-existing respiratory condition were associated with *mild* but not *severe* breathlessness. Individuals of Black ethnicity were less likely to report *mild* breathlessness. Participants aged 60 to 79 years were less likely to report *severe* breathlessness than the reference group.

Table S12 Multinomial logistic regression for PSQ breathlessness at the research visit

Dependent: Breathlessness at follow-up visit		OR (95% CI)	
		Mildly breathless	Severely breathless
Sex at birth	Male	1.00	1.00
	Female	1.23 (0.90-1.67)	1.81 (1.21-2.71)
Age at admission (years)	50-59	1.00	1.00
	<30	0.68 (0.22-2.10)	0.84 (0.23-3.12)
	30-39	0.94 (0.51-1.72)	0.28 (0.10-0.78)
	40-49	0.88 (0.55-1.40)	0.55 (0.30-1.01)
	60-69	0.99 (0.68-1.46)	0.53 (0.32-0.88)
	70-79	0.82 (0.50-1.35)	0.22 (0.10-0.47)
	80+	1.04 (0.44-2.44)	0.52 (0.17-1.61)
Index of Multiple Deprivation	5 - least deprived	1.00	1.00
	4	1.14 (0.71-1.80)	1.51 (0.69-3.31)
	3	1.07 (0.67-1.70)	2.52 (1.23-5.18)
	2	1.26 (0.81-1.97)	3.02 (1.49-6.12)
	1 - most deprived	1.39 (0.87-2.21)	4.82 (2.42-9.60)
Ethnicity	White	1.00	1.00
	South Asian	0.78 (0.48-1.26)	0.77 (0.40-1.51)
	Black	0.42 (0.22-0.81)	0.89 (0.45-1.75)
	Mixed	0.67 (0.25-1.81)	1.01 (0.33-3.07)
	Other	0.47 (0.21-1.08)	0.98 (0.38-2.51)
BMI (kg/m^2)	-	1.03 (1.01-1.06)	1.01 (0.98-1.04)
Number of comorbidities	-	1.09 (0.99-1.20)	1.26 (1.12-1.42)
Pre-existing respiratory condition	No	1.00	1.00
	Yes	1.54 (1.09-2.19)	1.54 (0.98-2.44)
Pre-existing depression or anxiety	No	1.00	1.00
	Yes	1.76 (1.10-2.81)	1.14 (0.62-2.09)
Admission duration (days)	-	1.00 (0.99-1.01)	1.02 (1.00-1.03)
WHO clinical progression scale	WHO – class 3-4	1.00	1.00
	WHO – class 5	0.87 (0.57-1.34)	0.89 (0.50-1.59)
	WHO – class 6	1.08 (0.67-1.75)	0.47 (0.23-0.98)
	WHO – class 7-9	1.18 (0.65-2.13)	1.06 (0.50-2.25)
The reference group (not shown) was the Not breathless group (n=675). Mildly breathless (n=364), Severely breathless (n=187)			

Sensitivity analyses: Dyspnoea 12 score at the research visit as a linear outcome
Figure S7 demonstrates the relationship between breathlessness reported at the research visit as recorded using the PSQ and Dyspnoea-12 scores.

Figure S7 Relationship of PSQ breathlessness and Dyspnoea 12 score collected at the research visit

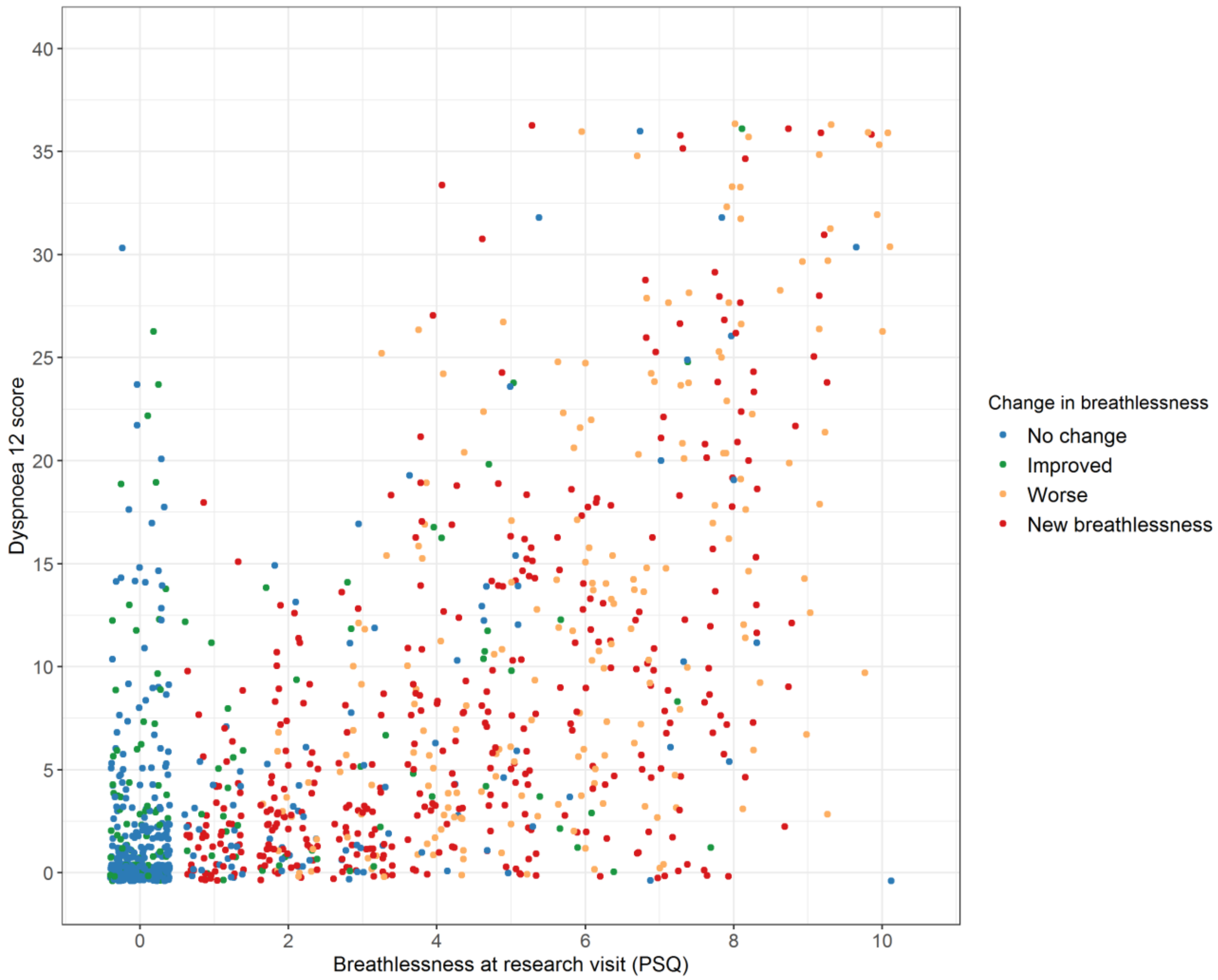

Table S13 Linear regression model - Dyspnoea 12 at the research visit

When breathlessness at the research visit, as recorded by Dyspnoea-12, was used as the dependent variable in a linear regression (Table S11), the most deprived quintile, female sex, admission duration, pre-existing respiratory condition, depression/anxiety and breathlessness prior to COVID-19 were significantly associated with a higher Dyspnoea-12 score. Participants 60 years or older were less likely to report breathlessness compared to the reference group.

Dependent: Dyspnoea-12 score		Value	Coefficient (univariable)	Coefficient (multivariable)
Sex at birth	Male	5.5 (7.9)	5.5 (7.9)	-
	Female	7.7 (8.9)	7.7 (8.9)	2.26 (1.27 to 3.25, p<0.001)
Age at admission (years)	50-59	7.5 (9.2)	-	-
	<30	6.0 (6.6)	-1.50 (-4.93 to 1.92, p=0.390)	-3.41 (-6.84 to 0.03, p=0.052)
	30-39	5.2 (7.5)	-2.25 (-4.36 to -0.15, p=0.036)	-2.59 (-4.70 to -0.49, p=0.016)
	40-49	5.6 (7.7)	-1.95 (-3.46 to -0.45, p=0.011)	-1.56 (-3.09 to -0.03, p=0.046)
	60-69	6.0 (7.9)	-1.49 (-2.74 to -0.24, p=0.020)	-2.14 (-3.43 to -0.85, p=0.001)
	70-79	5.5 (8.4)	-1.98 (-3.52 to -0.43, p=0.012)	-2.94 (-4.58 to -1.30, p<0.001)
	80+	4.8 (7.4)	-2.69 (-5.46 to 0.09, p=0.058)	-4.09 (-7.00 to -1.18, p=0.006)
Index of Multiple Deprivation	5 - least deprived	4.9 (6.7)	-	-
	4	4.6 (6.3)	-0.34 (-1.89 to 1.20, p=0.664)	0.18 (-1.40 to 1.75, p=0.825)
	3	6.5 (8.7)	1.62 (0.09 to 3.15, p=0.038)	1.32 (-0.24 to 2.87, p=0.097)
	2	6.4 (8.5)	1.50 (0.02 to 2.98, p=0.048)	1.13 (-0.40 to 2.66, p=0.148)
	1 - most deprived	8.1 (9.8)	3.23 (1.75 to 4.71, p<0.001)	3.13 (1.58 to 4.68, p<0.001)
Ethnicity	White	6.3 (8.2)	-	-
	South Asian	5.9 (8.7)	-0.46 (-1.93 to 1.01, p=0.540)	0.38 (-1.21 to 1.98, p=0.637)
	Black	7.5 (10.0)	1.15 (-0.74 to 3.03, p=0.233)	1.10 (-0.83 to 3.03, p=0.265)
	Mixed	4.8 (7.6)	-1.56 (-4.56 to 1.43, p=0.306)	-1.17 (-4.22 to 1.88, p=0.451)
	Other	4.8 (7.2)	-1.55 (-4.03 to 0.93, p=0.219)	-0.51 (-3.11 to 2.09, p=0.700)
BMI	[16.5,77.8]	6.2 (8.3)	0.17 (0.09 to 0.24, p<0.001)	0.26 (-0.14 to 0.65, p=0.201)
Number of comorbidities	[0.0,17.0]	6.2 (8.3)	0.87 (0.64 to 1.11, p<0.001)	0.11 (-0.22 to 0.43, p=0.525)
Pre-existing depression or anxiety	No	5.7 (8.0)	-	-
	Yes	9.7 (9.8)	4.06 (2.75 to 5.37, p<0.001)	2.05 (0.44 to 3.65, p=0.013)
Pre-existing respiratory condition	No	5.0 (7.3)	-	-
	Yes	9.7 (10.0)	4.70 (3.65 to 5.75, p<0.001)	2.46 (1.24 to 3.68, p<0.001)
Smoking	Never	5.9 (8.2)	-	-
	Ex-smoker	6.7 (8.4)	0.82 (-0.16 to 1.80, p=0.101)	0.34 (-0.68 to 1.36, p=0.513)
	Current smoker	10.4 (10.2)	4.58 (0.45 to 8.71, p=0.030)	3.69 (-0.73 to 8.12, p=0.102)
Breathlessness before admission (PSQ)	[0.0,10.0]	6.3 (8.4)	1.25 (1.02 to 1.47, p<0.001)	0.88 (0.60 to 1.15, p<0.001)
Admission duration (days)	[0.0,171.0]	6.3 (8.4)	0.02 (-0.00 to 0.05, p=0.081)	0.03 (0.00 to 0.06, p=0.034)
WHO clinical progression scale	WHO – class 3-4	6.8 (8.3)	-	-
	WHO – class 5	6.0 (8.2)	-0.80 (-2.17 to 0.57, p=0.250)	-0.95 (-2.36 to 0.46, p=0.185)
	WHO – class 6	6.4 (8.6)	-0.37 (-1.91 to 1.18, p=0.642)	-0.57 (-2.19 to 1.05, p=0.488)
	WHO – class 7-9	6.2 (8.4)	-0.61 (-2.13 to 0.92, p=0.436)	-1.06 (-2.99 to 0.87, p=0.280)

BMI = Body Mass Index. PSQ = Patient Symptom Questionnaire. World Health Organization (WHO) clinical progression scale: not requiring continuous supplemental oxygen (levels 3-4); continuous supplemental oxygen only (level 5); Continuous Positive Airway Pressure (CPAP) ventilation, Bi-Level Positive Airway Pressure or High Flow Nasal Oxygen (HFNO) (level 6); Invasive Mechanical Ventilation (IMV), Extra-Corporeal Membrane Oxygenation (ECMO) and acute Renal Replacement Therapy (RRT) (levels 7-9). The linear regression model was also adjusted for BMI^2.

Reporting guidelines

The manuscript was guided by the Strengthening the Reporting of Observational Studies in Epidemiology checklist (Table S3) [2] and the Transparent reporting of a multivariable prediction model for individual prognosis or diagnosis (Table S4) [3].

Table S14 STROBE Statement—Checklist of items that should be included in reports of cohort studies [2]

	Item.	Recommendation	Page No.
**Title and abstract**	1	(*a*) Indicate the study's design with a commonly used term in the title or the abstract	Title page
		(*b*) Provide in the abstract an informative and balanced summary	Page 1
**Introduction**			
Background/rationale	2	Explain the scientific background and rationale for the investigation being reported	Page 2
Objectives	3	State specific objectives, including any prespecified hypotheses	Page 2
**Methods**			
Study design	4	Present key elements of study design early in the paper	Page 2
Setting	5	Describe the setting, locations, and relevant dates, including periods of recruitment, exposure, follow-up, and data collection	Page 2-3
Participants	6	(*a*) Give the eligibility criteria, and the sources and methods of selection of participants. Describe methods of follow-up	Page 2
		(*b*) For matched studies, give matching criteria and number of exposed and unexposed	-
Variables	7	Clearly define all outcomes, exposures, predictors, potential confounders, and effect modifiers. Give diagnostic criteria, if applicable	Page 3
Data sources/ measurement	8*	For each variable of interest, give sources of data and details of methods of assessment (measurement). Describe comparability of assessment methods if more than one group	Page 3
Bias	9	Describe any efforts to address potential sources of bias	Page 8-9
Study size	10	Explain how the study size was arrived at	Page 2-3
Quantitative variables	11	Explain how quantitative variables were handled in the analyses. If applicable, describe which groupings were chosen and why	Page 3-4
Statistical methods	12	(*a*) Describe all statistical methods, including those used to control for confounding	Page 3-4
		(*b*) Describe any methods used to examine subgroups and interactions	Page 4
		(*c*) Explain how missing data were addressed	Page 4
		(*d*) If applicable, explain how loss to follow-up was addressed	-
		(*e*) Describe any sensitivity analyses	Page 3
**Results**			
Participants	13*	(a) Report numbers of individuals at each stage of study—e.g. numbers potentially eligible, examined for eligibility, confirmed eligible, included in the study, completing follow-up, and analysed	Page 4-5
		(b) Give reasons for non-participation at each stage	Page 2,4
		(c) Consider use of a flow diagram	Figure 1
Descriptive data	14*	(a) Give characteristics of study participants (eg demographic, clinical, social) and information on exposures and potential confounders	Page 5; Table 1-3
		(b) Indicate number of participants with missing data for each variable of interest	Page 4-5; Table 1-3
		(c) Summarise follow-up time (e.g., average and total amount)	Page 4
Outcome data	15*	Report numbers of outcome events or summary measures over time	Page 5
Main results	16	(*a*) Give unadjusted estimates and, if applicable, confounder-adjusted estimates and their precision (eg, 95% confidence interval). Make clear which confounders were adjusted for and why they were included	Page 5-6; Table 4
		(*b*) Report category boundaries when continuous variables were categorized	Page 5, Table 1
		(*c*) If relevant, consider translating estimates of relative risk into absolute risk for a meaningful time period	-
Other analyses	17	Report other analyses done—analyses of subgroups and interactions, and sensitivity analyses	Page 5-6
**Discussion**			
Key results	18	Summarise key results with reference to study objectives	Page 7
Limitations	19	Discuss limitations of the study, taking into account sources of potential bias or imprecision. Discuss both direction and magnitude of any potential bias	Page 8-9
Interpretation	20	Give a cautious overall interpretation of results considering objectives, limitations, multiplicity of analyses, results from similar studies, and other relevant evidence	Page 7-8
Generalisability	21	Discuss the generalisability (external validity) of the study results	Page 8
**Other information**			
Funding	22	Give the source of funding and the role of the funders for the present study and, if applicable, for the original study on which the present article is based	Page 10

Table S15 TRIPOD reporting guidelines checklist [3]

Domain		Reporting Item	Page Number
**Title**			
	1	Identify the study as developing and / or validating a multivariable prediction model, the target population, and the outcome to be predicted.	Title
**Abstract**			
	2	Provide a summary of objectives, study design, setting, participants, sample size, predictors, outcome, statistical analysis, results, and conclusions.	Page 1
**Introduction**			
	3a	Explain the medical context (including whether diagnostic or prognostic) and rationale for developing or validating the multivariable prediction model, including references to existing models.	Page 2
	3b	Specify the objectives, including whether the study describes the development or validation of the model or both.	Page 2
**Methods**			
Source of data	4a	Describe the study design or source of data (e.g., randomized trial, cohort, or registry data), separately for the development and validation data sets, if applicable.	Page 2-3
Source of data	4b	Specify the key study dates, including start of accrual; end of accrual; and, if applicable, end of follow-up.	Page 2-3
Participants	5a	Specify key elements of the study setting (e.g., primary care, secondary care, general population) including number and location of centres.	Page 2-3
Participants	5b	Describe eligibility criteria for participants.	Page 2-3
Participants	5c	Give details of treatments received, if relevant	Page 2-3
Outcome	6a	Clearly define the outcome that is predicted by the prediction model, including how and when assessed.	Page 4
Outcome	6b	Report any actions to blind assessment of the outcome to be predicted.	-
Predictors	7a	Clearly define all predictors used in developing or validating the multivariable prediction model, including how and when they were measured	Page 4
Predictors	7b	Report any actions to blind assessment of predictors for the outcome and other predictors.	-
Sample size	8	Explain how the study size was arrived at.	Page 2-3
Missing data	9	Describe how missing data were handled (e.g., complete-case analysis, single imputation, multiple imputation) with details of any imputation method.	Page 4
Statistical analysis methods	10a	If you are developing a prediction model describe how predictors were handled in the analyses.	Page 4
Statistical analysis methods	10b	If you are developing a prediction model, specify type of model, all model-building procedures (including any predictor selection), and method for internal validation.	Page 4
Statistical analysis methods	10c	If you are validating a prediction model, describe how the predictions were calculated.	-
Statistical analysis methods	10d	Specify all measures used to assess model performance and, if relevant, to compare multiple models.	Page 4
Statistical analysis methods	10e	If you are validating a prediction model, describe any model updating (e.g., recalibration) arising from the validation, if done	-
Risk groups	11	Provide details on how risk groups were created, if done.	-
Development vs. validation	12	For validation, identify any differences from the development data in setting, eligibility criteria, outcome, and predictors.	-
**Results**			
Participants	13a	Describe the flow of participants through the study, including the number of participants with and without the outcome and, if applicable, a summary of the follow-up time. A diagram may be helpful.	Page 4-5<br>Figure 1
Participants	13b	Describe the characteristics of the participants (basic demographics, clinical features, available predictors), including the number of participants with missing data for predictors and outcome.	Page 4-5<br>Table 1-3
Participants	13c	For validation, show a comparison with the development data of the distribution of important variables (demographics, predictors and outcome).	-
Model development	14a	If developing a model, specify the number of participants and outcome events in each analysis.	Page 4,<br>Table 4
Model development	14b	If developing a model, report the unadjusted association, if calculated between each candidate predictor and outcome.	Table 4
Model specification	15a	If developing a model, present the full prediction model to allow predictions for individuals (i.e., all regression coefficients, and model intercept or baseline survival at a given time point).	Table 4,<br>Equation E1
Model specification	15b	If developing a prediction model, explain how to the use it.	Equation S1
Model performance	16	Report performance measures (with CIs) for the prediction model.	Page 6

Model-updating	17	If validating a model, report the results from any model updating, if done (i.e., model specification, model performance).	-
**Discussion**			
Limitations	18	Discuss any limitations of the study (such as non-representative sample, few events per predictor, missing data).	Page 8-9
Interpretation	19a	For validation, discuss the results with reference to performance in the development data, and any other validation data	-
Interpretation	19b	Give an overall interpretation of the results, considering objectives, limitations, results from similar studies, and other relevant evidence.	Page 7-8
Implications	20	Discuss the potential clinical use of the model and implications for future research	-
**Other information**			
Supplementary information	21	Provide information about the availability of supplementary resources, such as study protocol, Web calculator, and data sets.	Page 10-12
Funding	22	Give the source of funding and the role of the funders for the present study.	Page 10

References

S1 WHO Working Group on the Clinical Characterisation and Management of COVID-19 infection. A minimal common outcome measure set for COVID-19 clinical research. *Lancet Infect Dis*. 2020;20(8):e192-197.

S2 Von Elm E, Altman DG, Egger M, et al. The Strengthening the Reporting of Observational Studies in Epidemiology (STROBE) statement: guidelines for reporting observational studies. *Ann Intern Med*. 2007;147(8):573-577.

S3 Moons KG, Altman DG, Reitsma JB et al. Transparent Reporting of a multivariable prediction model for Individual Prognosis or Diagnosis (TRIPOD): explanation and elaboration. Ann Intern Med. 2015;162(1):W1-73.
